# Integrating BSA-Seq and RNA-Seq to Identify Major QTLs and Candidate Genes Conferring Resistance to Fusarium Ear Rot in Maize

**DOI:** 10.3390/plants15060985

**Published:** 2026-03-23

**Authors:** Shufeng Sun, Jie Xu, Jiaxin Huang, Yuying Fan, Gongjian Li, Zhuanfang Hao, Jianfeng Weng, Zhennan Xu, Xinhai Li

**Affiliations:** 1 College of Agronomy, Shenyang Agricultural University, Dongling Street, Shenhe District, Shenyang 110866, China; 2 National Nanfan Research Institute, Chinese Academy of Agricultural Sciences, Sanya 572000, China; 3 State Key Laboratory of Crop Gene Resources and Breeding, Institute of Crop Sciences, Chinese Academy of Agricultural Sciences, Beijing 100081, China

**Keywords:** FER, *F. verticillioides*, combined analysis, QTL

## Abstract

*Fusarium* ear rot (FER), caused by *Fusarium verticillioides*, is a devastating disease that substantially reduces maize yield and compromises kernel quality. To investigate the genetic and molecular basis of resistance, an F_2_ population derived from a cross between the resistant inbred line 3IBZ2 and the susceptible inbred line KW5G321 was analysed. By integrating bulked segregant analysis sequencing (BSA-Seq) with RNA sequencing (RNA-Seq), a major quantitative trait locus (QTL), designated *qFER4*, was identified on chromosome 4. Genetic analysis further demonstrated that *qFER4* confers resistance through partial dominance. Transcriptome profiling of the resistant line revealed 7684 and 7906 differentially expressed genes (DEGs) at 36 and 72 h post inoculation (hpi), respectively. These DEGs were significantly enriched in defence-related biological processes and pathways, including phenylpropanoid biosynthesis, jasmonic acid signalling, MAPK cascades, and plant-pathogen interactions. By combining QTL mapping with transcriptome analyses, four candidate genes within the *qFER4* interval were screened. Sequence analysis identified extensive structural variations in the promoter and coding regions of *Zm00001d053393*, including a premature stop codon predicted to lead to a gain-of-function mutation. In contrast, the other three genes exhibited only minor promoter polymorphisms with identical coding sequences between the parental lines. Overall, this study identifies a novel major-effect QTL and candidate gene associated with FER resistance, providing a foundation for gene function and a valuable genetic resource for breeding FER-resistant maize varieties.

## 1. Introduction

Maize (*Zea mays* L.) is a globally important cereal crop that serves as a staple food, a major component of animal feed, and a key raw material for bioenergy and industrial processing. However, frequent outbreaks of fungal diseases have become a major constraint to stable and high-yield production across global growing regions [1,2]. Among these diseases, stalk rot and ear rot caused by *Fusarium* species are particularly destructive, leading to severe yield losses and deterioration of grain quality. In addition to physical damage, *Fusarium* species produce a range of mycotoxins that pose substantial risks to human and animal health [3,4]. In recent years, FER, primarily caused by *F. verticillioides*, has shown an increasing prevalence in China. For instance, average FER incidence reached 42.9% in the Huanghuaihai region in 2004 [5] and 63.6% in Gansu Province in 2009 [6]. Similar epidemics have been documented globally in countries including Mexico and Argentina [7,8]. The rising incidence and severity of FER highlight the urgent need to identify resistant varieties and clone resistance genes to achieve sustainable maize production.

In recent years, research groups worldwide have employed diverse phenotyping approaches to evaluate maize germplasm from a wide range of ecological regions and genetic backgrounds for ear rot resistance. These efforts have revealed that the resistant germplasm remains largely confined to certain regions of the Americas and Europe, including the United States, Canada, Mexico, Argentina, and Brazil [9,10]. Moreover, highly resistant germplasm represents less than 10% of the screened germplasm. Most commercial inbred lines and hybrids exhibit susceptibility or moderate susceptibility to ear rot, thereby limiting the diversity of available resistance sources in China [11]. Adding to the challenge, the genetic diversity of physiological races among ear rot pathogens further complicates the development of durable resistance [12,13]. Since the 1990s, numerous quantitative trait loci (QTLs) associated with FER have been identified, distributed across chromosomes 1–10. Subsequent studies have further mapped multiple resistance-related QTLs and candidate genes. However, most QTLs identified from resistant inbred lines are characterized by minor effects and high environmental sensitivity, and practical molecular markers closely linked or co-segregating with these QTLs remain unavailable [14]. Furthermore, the maize ear rot is influenced by multiple factors, including pathogen species, planting density, and environmental conditions, which further complicates resistance evaluation and limits the direct application of most identified QTLs in breeding programs.

Although researchers have identified multiple resistance-related QTLs in maize, only a limited number of resistance genes against *Fusarium* diseases have been successfully cloned to date. Using linkage mapping, Ye et al. [15] identified *ZmAuxRP1* as the causal gene underlying the major QTL *qRfg2*, which confers resistance to *F. graminearum*-induced stalk rot by regulating auxin signalling and transport pathways. Similarly, Ma et al. [16] identified *ZmXYXT2* through linkage-based QTL mapping of a recombinant inbred line (RIL) population. *ZmXYXT2* enhances physical barriers against fungal invasion by modulating cell wall composition and thickness. Via association mapping, Dong et al. [17] identified *ZmGAE1*, encoding a UDP-D-glucuronate 4-epimerase, as a negative regulator of resistance to FER; variations in its promoter were shown to improve resistance and reduce fumonisin (FUM) accumulation. Moreover, Ma et al. [18] demonstrated that natural variation in *ZmWAX2* contributes to quantitative resistance against *F. verticillioides* by modulating epicuticular wax deposition. Mutant-based approaches have also provided valuable functional evidence: CRISPR/Cas9-mediated knockout of *ZmFER1* confers strong FER resistance without sacrificing grain yield [19], while disruption of the lipoxygenase gene *ZmLOX3* attenuates fungal colonisation and suppresses FUM production [20,21]. Although a subset of disease resistance genes has been cloned in maize, FER resistance remains a quantitative trait governed primarily by genes with minor effects. Therefore, the cloning of novel resistance loci and the strategic pyramiding of their favorable alleles offer a promising path for developing durably resistant maize varieties.

To elucidate the genetic basis of resistance to FER and clone novel resistant loci, we performed BSA-Seq, which identified a major *QTL_qFER4* with strong and consistent association signals. To further characterise the transcriptional responses to infection, RNA-Seq was performed on kernel tissues at 0, 36, and 72 h post-inoculation (hpi). By integrating genetic mapping with transcriptomic profiles, we identified a high-confidence candidate gene within the *qFER4* interval. Subsequent validation by RT-qPCR and comparative sequence analyses further pinpointed *Zm00001d053393* as the most likely candidate gene underlying *qFER4*.

## 2. Results

### 2.1. Phenotypic Analysis of Parental Lines for Resistance to FER

**Figure 1:**
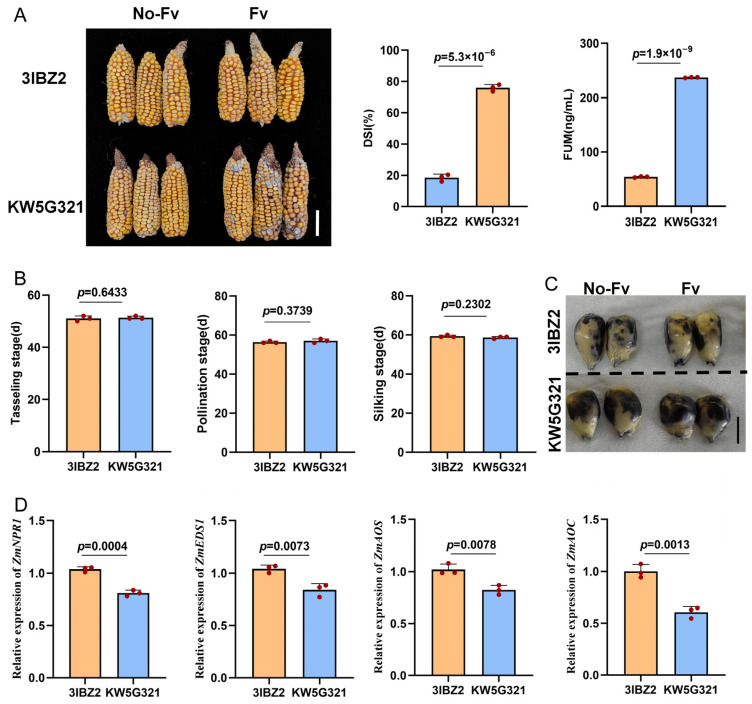
Phenotypes of 3IBZ2 and KW5G321. (**A**) Phenotypes of 3IBZ2 and KW5G321 ears without inoculation and after inoculation with *F. verticillioides*. Scale bar = 5 cm; DSI of resistant (3IBZ2) and susceptible materials (KW5G321); FUM content measurement. (**B**) Tasseling, pollination, and silking stages of 3IBZ2 and KW5G321. (**C**) Schematic diagram of NBT reactive oxygen species staining. Scale bar = 0.5 cm. (**D**) Relative expression levels of SA marker genes; Relative expression levels of JA marker genes.

The resistant inbred line 3IBZ2 exhibited strong resistance to FER, with an average disease severity index (DSI) of 18.60%, whereas the susceptible inbred line KW5G321 displayed severe symptoms, with an average DSI of 75.98% (*p* = 5.3 × 10^−6^) (Figure 1A). Correspondingly, FUM content was significantly lower in 3IBZ2 than in KW5G321 (*p* = 1.9 × 10^−9^) (Figure 1A). To rule out the influence of phenological variation on susceptibility, we compared key flowering traits (tasseling, pollen shed, and silking) between the two lines and observed no significant differences (Figure 1B), indicating that the resistance was independent of flowering time. Pathogenic fungi can trigger oxidative stress responses in plants. To assess whether differential oxidative stress correlates with the contrasting FER phenotypes, we compared reactive oxygen species (ROS) accumulation in kernels of 3IBZ2 and KW5G321 at 25 days after pollination using NBT staining. The resistant line 3IBZ2 exhibited sparse and faint blue precipitates, indicating low ROS levels, whereas KW5G321 showed dense and intense staining patterns, reflecting pronounced oxidative stress (Figure 1C). To further elucidate the underlying defence mechanisms, we analysed the expression of key signalling genes in the salicylic acid (SA) and jasmonic acid (JA) pathways. RT-qPCR analysis demonstrated significant upregulation of key salicylic acid (*ZmNPR1* and *ZmEDS1*) and jasmonic acid (*ZmAOS* and *ZmAOC*) signalling genes in the resistant line 3IBZ2 compared to the susceptible line KW5G321 (*p* < 0.05) (Figure 1D). These findings indicate that the observed reduction in oxidative damage is functionally associated with the simultaneous activation of both SA and JA-mediated defence pathways, which together enhance resistance to FER.

### 2.2. Phenotypic Evaluation of the F_2_ Population for Resistance to FER

**Figure 2:**
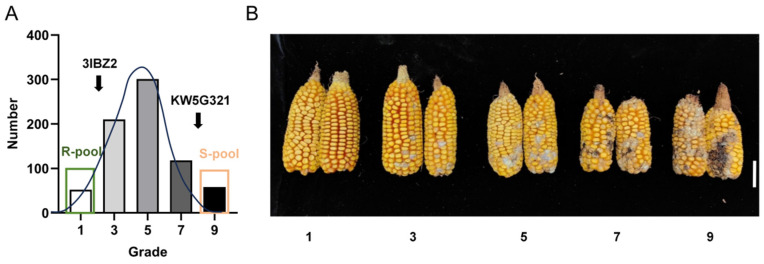
Phenotypic analysis and evaluation of maize FER-resistant populations. (**A**) Probability distribution of ear rot of 739 maize plants in the F_2_ population. (**B**) Phenotypic evaluation criteria for the Population. Scale bar = 5 cm.

To elucidate the genetic basis of resistance to FER, 739 F_2_ individuals derived from a cross between the resistant line 3IBZ2 and the susceptible line KW5G321 were evaluated under field inoculation conditions. As expected, FER symptoms across the F_2_ population exhibited extensive phenotypic variation, ranging from nearly symptomless ears to severe ear rot symptoms comparable to those of the susceptible parent (Figure 2A). Statistical analysis revealed that the DSI among the F_2_ population followed an approximately continuous distribution (Figure 2B). The DSI ranged from 24.39% to 86.10%, with a population mean of 51.42%. Calculated kurtosis (−0.236) and skewness (0.269) indicated a near-normal distribution without significant deviation (Table 1). These results suggest that FER resistance in this population is a quantitative trait likely controlled by multiple loci with differing effect sizes. Furthermore, the presence of highly resistant individuals (score = 1) and extremely susceptible individuals (score = 9) was observed within the population, confirming the presence of transgressive segregation. This phenotypic variation supported the construction of extreme phenotype bulks for subsequent BSA-Seq analysis.

### 2.3. BSA-Seq Identifies Major QTLs for FER

**Figure 3:**
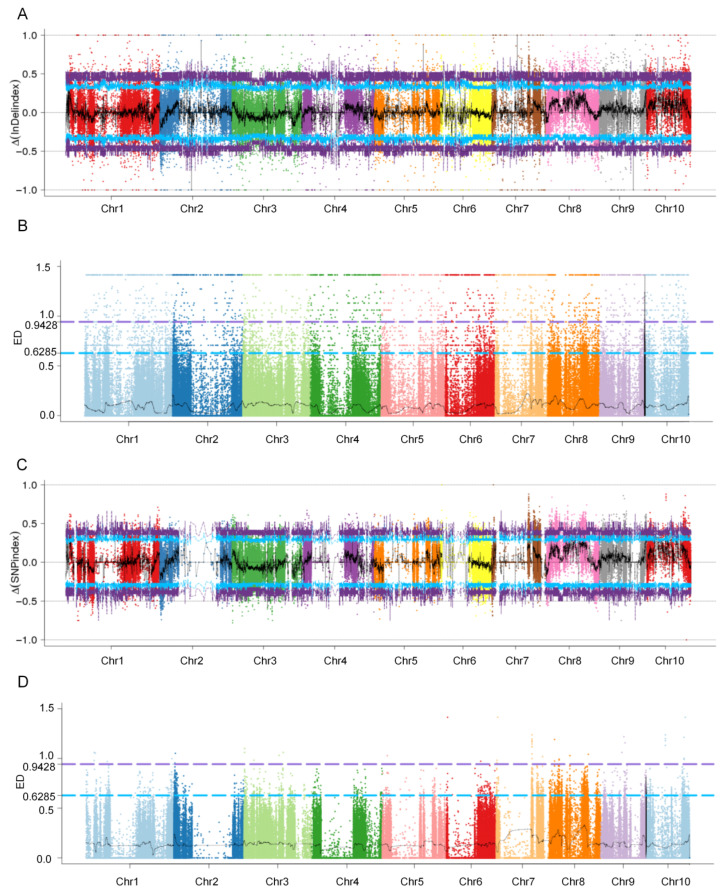
Quantitative trait locus (QTL) analysis of maize ear rot using two QTL-seq methods. (**A**) Manhattan plot showing the distribution of ∆ (indel index) on chromosomes. (**B**) Manhattan plot showing the distribution of indel ED on chromosomes. (**C**) Manhattan plot showing the distribution of ∆ (SNP index) on chromosomes. (**D**) Manhattan plot showing the distribution of SNP ED on chromosomes. Blue and purple lines represent 95 and 99% confidence intervals, respectively, and black lines represent the fitted line, which was drawn using sliding window analysis. Numbers on the horizontal coordinates represent chromosome numbers.

To identify genomic regions associated with resistance to FER, the BSA-seq approach was performed using DNA pooled from the two parental lines and two extreme phenotype bulks. The clean reads were aligned to the B73 reference genome, resulting in the identification of 35,992,006 single-nucleotide polymorphisms (SNPs) and 3,524,617 insertions/deletions (InDels) (Supplementary Tables S2 and S3). Homozygous polymorphic sites between the two parental lines were subsequently selected based on genotyping data, yielding high-quality 201,127 polymorphic SNPs and 238,585 polymorphic InDels for BSA-seq. Putative quantitative trait loci (QTLs) linked to FER resistance were detected using the Δ(SNP index), Δ(InDel index) and ED methods (Figure 3A–D).

Based on the Δ(InDel index) analysis, seven genomic intervals surpassing the 95% significance threshold were detected on chromosomes 4, 6, 7, 8, and 10, with physical lengths of: *qFER4* (227.4–230.1 Mb, 2.7 Mb); *qFER6* (30.5–31.7 Mb, 1.2 Mb); *qFER7* (84.2–85.5 Mb, 1.3 Mb); *qFER8.1* (93.2–94.9 Mb, 1.7 Mb); *qFER10.1* (32.4–36.3 Mb, 3.9 Mb); *qFER10.2* (55.5–59.5 Mb, 4.0 Mb); and *qFER10.3* (94.4–98.0 Mb, 3.6 Mb) (Table 2). Similarly, the Δ(SNP index) analysis identified six significant intervals surpassing the 95% threshold, located on chromosomes 8, 9, and 10, with the following physical positions: *qFER8.2* (79.6–88.7 Mb, 9.1 Mb); *qFER8.3* (113.8–116.7 Mb, 2.9 Mb); *qFER8.4* (126.3–131.7 Mb, 5.4 Mb); *qFER9* (156.9–158.9 Mb, 2.0 Mb); *qFER10.4* (103.7–105.5 Mb, 1.8 Mb); and *qFER10.5* (113.3–117.8 Mb, 4.5 Mb). According to the ED analysis, the highest ED peak was the region *qFER4* (227.4–230.1 Mb) with ED values ranging from 1.32 to 1.41 (Table 2). Taking into account the concordance across methods and the magnitude of the ED peak, *qFER4* was prioritised as the most promising candidate intervals for the identification of genes conferring resistance to FER in maize.

### 2.4. qFER4-Mediated Genetic Model for Resistance to FER

**Figure 4:**
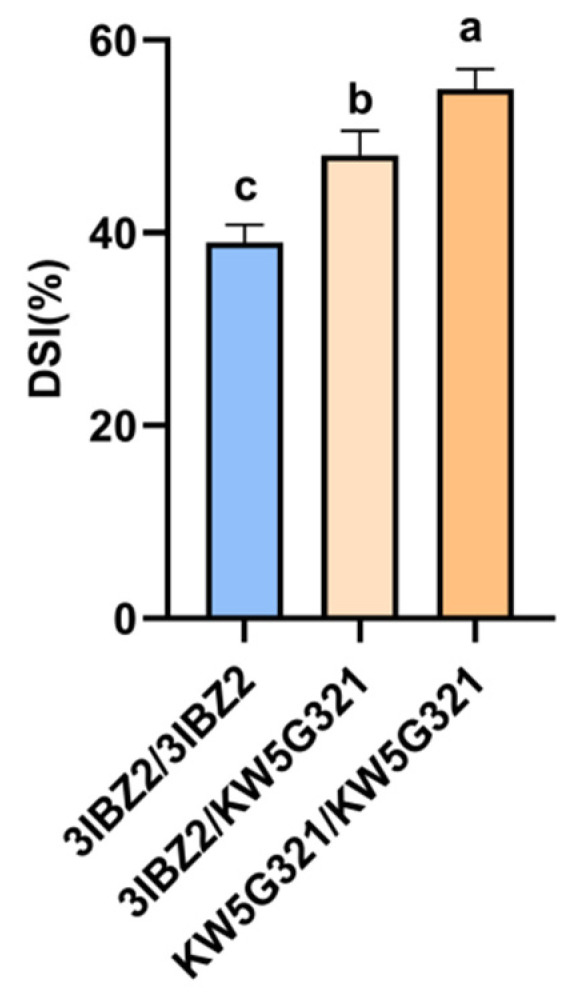
Genetic model of qFER4 action based on F_2_ populations. According to genotypes within the qFER4 region between markers InDel-1 and InDel-2, the F_2_ populations were divided into three genotypes (3IBZ2/3IBZ2, 3IBZ2/KW5G321, and KW5G321/KW5G321) in 2024. The average disease severity index (%) values are shown. a, b, c indicate significant differences at the *p* < 0.05. Error bars indicate standard errors of the means.

To elucidate the genetic effects of the *qFER4* locus on FER resistance, an F_2_ population derived from the resistant 3IBZ2 and susceptible parent KW5G321was evaluated. Within the *qFER4* interval, two polymorphic InDel markers (InDel-1 and InDel-2) were developed to genotype individuals, classifying them into three groups: 3IBZ2/3IBZ2 (homozygous), 3IBZ2/KW5G321 (heterozygous), and KW5G321/KW5G321 (homozygous). A total of 739 F_2_ plants were evaluated for FER resistance in Sanya. DSI analysis revealed that homozygous plants for the resistant allele (3IBZ2/3IBZ2) exhibited a significantly lower DSI (38.95%) than those homozygous for the susceptible allele (KW5G321) (54.92%). Heterozygous individuals (3IBZ2/KW5G321) displayed an intermediate DSI of 48.04%, which was statistically positioned between the two homozygous groups (Figure 4). These results indicate that the resistance conferred by *qFER4* is inherited with partial dominance, highlighting its important role in the genetic regulation of FER resistance in maize.

### 2.5. Identification of Candidate Genes Within the QTL Intervals

**Figure 5:**
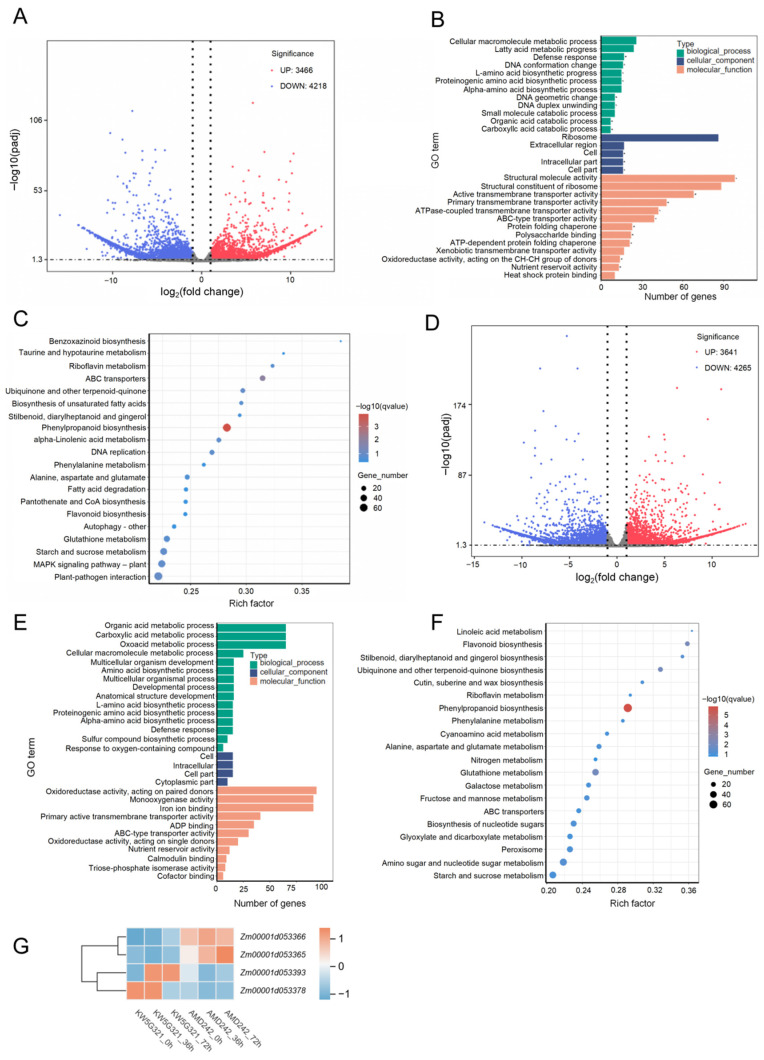
Number of differentially expressed genes (DEGs) identified by RNA-seq analysis. (**A**) Volcano map showing the DEGs at 36 hpi between 3IBZ2 and KW5G321. (**B**) Gene Ontology (GO) enrichment analysis of DEGs at 36 hpi between 3IBZ2 and KW5G321. (**C**) Differential gene KEGG enrichment analysis at 36 hpi. (**D**) Volcano map showing the DEGs at 72 hpi between 3IBZ2 and KW5G321. (**E**) Gene Ontology (GO) enrichment analysis of DEGs at 72 hpi between 3IBZ2 and KW5G321. (**F**) Differential gene KEGG enrichment analysis at 72 hpi. (**G**) Heatmap of candidate gene at 0, 36 and 72 hpi between 3IBZ2 and KW5G321.

To elucidate the regulatory pathways and candidate genes involved in the control of FER in parents, we performed RNA-seq analysis on kernels of the resistant inbred line 3IBZ2 and the susceptible line KW5G321 at 0, 36, and 72 h post-inoculation (hpi) with *F. verticillioides*. At 36 hpi, comparative transcriptome analysis identified 7684 DEGs between 3IBZ2 and KW5G321, including 3466 upregulated and 4218 downregulated genes in 3IBZ2 relative to KW5G321 (Figure 5A). GO enrichment analysis showed that the upregulated genes in 3IBZ2 were significantly enriched in categories related to defence response and cellular macromolecule metabolic processes (Figure 5B). Consistently, KEGG pathway enrichment indicated that these DEGs were predominantly enriched in key defence-related pathways, including phenylpropanoid biosynthesis, α-linolenic acid metabolism, phenylalanine metabolism, the MAPK signalling pathway-plant, and plant-pathogen interaction (Figure 5C). At 72 hpi, a total of 7906 DEGs were identified between 3IBZ2 and KW5G321, including 3641 upregulated and 4265 downregulated genes in 3IBZ2 (Figure 5D). GO enrichment analysis showed that the upregulated DEGs were predominantly involved in defence responses, organic and carboxylic acid metabolic processes, oxoacid metabolism, responses to oxygen-containing compounds, and cellular macromolecule metabolic processes (Figure 5E). Consistently, KEGG pathway enrichment analysis demonstrated significant enrichment of these DEGs in phenylpropanoid biosynthesis, linoleic acid metabolism, phenylalanine metabolism, flavonoid biosynthesis, and ubiquinone and other terpenoid-quinone biosynthesis pathways (Figure 5F).

**Figure 6:**
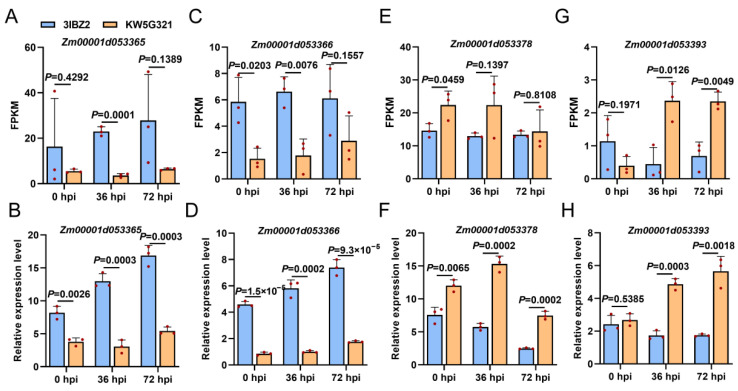
Expression analysis of candidate genes in response to *F. verticillioides*. (**A**) FPKM of *Zm00001d053365*. (**B**) Relative expression level of *Zm00001d053365*. (**C**) FPKM of *Zm00001d053366*. (**D**) Relative expression level of *Zm00001d053366*. (**E**) FPKM of *Zm00001d053378*. (**F**) Relative expression level of *Zm00001d053378*. (**G**) FPKM of *Zm00001d053393*. (**H**) Relative expression level of *Zm00001d053393* in 0, 36 and 72 hpi.

Based on the identified candidate QTL intervals, gene annotation within each interval was retrieved from the MaizeGDB database (http://www.maizegdb.org (accessed on 6 August 2025)) using the B73 reference genome (Zm-B73-REFERENCE-NAM-4.0). Within the *qFER4* interval, 36 genes were identified, among which four DEGs were detected at 36 hpi and two DEGs at 72 hpi (Supplementary Table S4). At 36 hpi, the DEGs located in *qFER4* included *Zm00001d053365*, *Zm00001d053366*, *Zm00001d053378*, and *Zm00001d053393*. Among them, *Zm00001d053365* and *Zm00001d053366* were upregulated in the resistant inbred line 3IBZ2, while *Zm00001d053378* and *Zm00001d053393* were downregulated in the susceptible inbred line KW5G321 (Figure 5G). At 72 hpi, the DEGs identified were *Zm00001d053365* and *Zm00001d053393*; of these, *Zm00001d053365* was upregulated in the resistant inbred line 3IBZ2, whereas *Zm00001d053393* was downregulated in the susceptible inbred line KW5G321 (Figure 5G). Notably, *Zm00001d053365* and *Zm00001d053393* showed consistent differential expression at both 36 and 72 h post-inoculation, indicating their potential critical roles in the resistance response to *F. verticillioides*. To validate the reliability of the RNA-seq data, we examined the expression patterns of four candidate genes (*Zm00001d053365*, *Zm00001d053366*, *Zm00001d053378*, *Zm00001d053393*) via RT-qPCR (Figure 6). The expression profiles of the four genes were strongly consistent with their corresponding FPKM values derived from transcriptome analysis. Specifically, *Zm00001d053365* and *Zm00001d053366* consistently showed higher expression in 3IBZ2 than KW5G321 across all time points. The *Zm00001d053378* and *Zm00001d053393* were expressed at lower levels in 3IBZ2 relative to KW5G321. Collectively, our transcriptomic profiling elucidated dynamic defence pathways activated upon *F. verticillioides* infection and pinpointed key candidate genes within a major resistance QTL.

### 2.6. Sequence Analysis of Candidate Genes in the qFER4

Based on the RNA-seq and RT-qPCR analysis, we analysed the coding sequences (CDS) and promoter sequences of the four candidate genes (*Zm00001d053365*, *Zm00001d053366*, *Zm00001d053378*, and *Zm00001d053393*) located within the *qFER4* interval on chromosome 4. In *Zm00001d053365*, the promoter region of 3IBZ2 contained 17 single-nucleotide polymorphisms (SNPs), one single-base insertion, and one five-base deletion compared to KW5G321, whereas no sequence variation was detected in its CDS or encoded protein (Supplementary Figure S1A–C). Similarly, the promoter of *Zm00001d053366* harboured one single-base insertion, one SNP, and one single-base deletion, while its CDS and protein sequences remained conserved between the two lines (Supplementary Figure S1D–F). For *Zm00001d053378*, four SNPs were identified in the promoter region, but its CDS and predicted protein sequence showed no detectable differences (Supplementary Figure S1G–I). In contrast, *Zm00001d053393* exhibited extensive structural variation. Its promoter region contained numerous SNPs and indels. Additionally, 14 SNPs and a single-base insertion were present in the first exon, and one single-base insertion and one SNP were detected in the second exon in 3IBZ2, collectively introducing a premature stop codon in the predicted protein sequence compared to KW5G321 (Supplementary Figure S1J–L). The findings above suggest that *Zm00001d053393* may represent a strong candidate gene underlying *qFER4* and could play a key regulatory role in FER resistance.

## 3. Discussion

FER, primarily caused by *F. verticillioides*, is a globally prevalent disease that leads to significant yield losses and contaminates grains with hazardous mycotoxins, thereby posing a dual threat to maize productivity and food safety. To combat FER, the identification of genetic resistance through quantitative trait locus (QTL) mapping has long been a major research focus [22,23]. Although numerous FER resistance QTLs have been reported across the maize genome, most exhibit only minor phenotypic effects, high environmental sensitivity, and a lack of closely linked molecular markers suitable for breeding applications, which limits their practical utility [14]. The existing resistance QTLs are primarily derived from tropical germplasm, such as CML171 and Qi319 [24,25]. Consequently, discovering major-effect QTLs from novel and stable resistance sources remains a pivotal yet challenging objective in maize disease resistance breeding. In this study, both the resistant parent 3IBZ2 and the susceptible parent KW5G321 were identified as temperate inbred lines, which enhances their applicability in breeding programs. The resistant line exhibits strong resistance. Using BSA-seq technology, we identified a novel resistance QTL that does not overlap with previously reported resistance QTLs [26].

The primary objective of this study was to identify such genetic resources. Through multi-environment field evaluations of hundreds of maize germplasm accessions over multiple years, we selected two inbred lines exhibiting extreme phenotypic differences in FER resistance. The resistant line, 3IBZ2, displayed a consistently high level of resistance across diverse environmental conditions, with a mean disease severity index (DSI) of only 18.60% and significantly lower FUM content in kernels (Figure 1A). In striking contrast, the susceptible line KW5G321 exhibited severe disease symptoms, with a mean DSI of 75.98%, representing a highly significant difference between the two lines (*p* = 5.3 × 10^−6^) (Figure 1A). Further physiological and biochemical analyses revealed that the resistant line 3IBZ2 accumulated lower levels of reactive oxygen species (ROS) following inoculation, while key genes involved in salicylic acid (SA) and jasmonic acid (JA) signaling pathways were significantly upregulated (Figure 1D), suggesting that its resistance may be associated with the early activation of hormone signaling networks. These marked differences in both phenotypic and physiological responses provided an ideal genetic framework for dissecting the genetic basis of FER resistance. Using an F_2_ segregating population of 739 individuals derived from the cross between 3IBZ2 and KW5G321, we employed bulked segregant analysis coupled with sequencing (BSA-seq)—a strategy that has been proven effective for rapid QTL mapping in various crop species [27,28]. Comprehensive analysis based on two complementary algorithms, Δ(SNP-index) and Euclidean distance (ED), identified a major QTL on chromosome 4, designated qFER4, spanning a physical interval of 227.4–230.1 Mb (Table 2). This locus exhibited strong association signals in both analytical methods, with ED values reaching 1.32–1.41—the highest among all detected QTLs—indicating its central role in mediating resistance. While previous studies on maize FER resistance have predominantly reported numerous small-effect QTLs distributed across multiple chromosomes, the *qFER4* interval identified in this study represents a novel major-effect resistance locus.

Following the identification of a major-effect QTL, elucidating its mode of inheritance is essential for evaluating its potential in breeding programs. Whether the genetic model is additive, dominant, or partially dominant directly determines selection strategies and the efficiency of allele introgression into elite germplasm [29]. In this study, we genotyped 739 F_2_ individuals using InDel markers (InDel-1 and InDel-2) developed within the *qFER4* interval and performed genotype–phenotype association analysis based on field disease severity data. The results revealed a consistent dose-dependent pattern: plants homozygous for the resistant allele (3IBZ2 type) exhibited the lowest disease severity (DSI = 38.95%), while those homozygous for the susceptible allele (KW5G321 type) showed the highest disease severity (DSI = 54.92%), with a statistically significant difference between the two groups. Heterozygous individuals (3IBZ2/KW5G321) displayed intermediate disease severity (DSI = 48.04%) (Figure 4), which differed significantly from both homozygous groups. The results of this study are similar to those of *QTL-qRfg2*, both indicating semi-dominance, suggesting that resistance QTLs derived from tropical and temperate inbred lines have comparable genetic effects [15]. These findings clearly demonstrate that the resistance conferred by *qFER4* follows a partially dominant mode of inheritance. This genetic model holds considerable practical value for breeding. The presence of a single favourable allele can confer a measurable level of resistance in progeny, thereby reducing the selection pressure required to achieve genome-wide homozygosity and enabling effective selection of resistance allele-carrying individuals in early generations.

Building upon the genetic localisation of a major-effect QTL, the functional characterisation of resistance mechanisms represents the logical next step for translating genetic discoveries into breeding applications [29]. RNA-seq has become an indispensable tool in this process, moving beyond static genetic maps to dynamically capture the global gene expression reprogramming triggered by pathogen attack [30]. This approach not only directly links genotype to phenotype by revealing the activated defence pathways but also serves as a powerful strategy for mining novel candidate genes and regulatory networks underlying complex traits [31,32]. In the context of quantitative disease resistance, where individual QTLs often encompass numerous genes, transcriptomic profiling of contrasting genotypes following infection provides critical functional evidence to pinpoint the key drivers within a genetic interval [33]. Here, to decipher the molecular basis of the resistance conferred by the novel locus *qFER4* in maize inbred line 3IBZ2, we performed a comparative transcriptomic analysis of 3IBZ2 and a susceptible control upon *F. verticillioides* infection.

Gene Ontology and KEGG enrichment analyses revealed that genes upregulated in the resistant line 3IBZ2 upon F. verticillioides infection were significantly enriched in core defense pathways at both 36 and 72 hpi, including phenylpropanoid biosynthesis, α-linolenic acid metabolism (JA biosynthesis), MAPK signaling, and plant-pathogen interaction-findings validated by RT-qPCR showing elevated JA marker genes in 3IBZ2. Integrating these transcriptomic data with QTL mapping, we focused on the *qFER4* interval and identified four differentially expressed genes, with *Zm00001d053365* and *Zm00001d053393* consistently differentially expressed at both time points, suggesting their core roles in resistance. Collectively, these results demonstrate that resistance in 3IBZ2 is orchestrated through an integrated, multi-layered defense network converging secondary metabolism (phenylpropanoids), defense hormone signaling (JA), enhanced pathogen perception and signal transduction (MAPK cascades), and key responsive genes within a major-effect QTL, providing a unified mechanistic basis for its enhanced resistance to *F. verticillioides*.

This analysis aimed to define the specific defence pathways mobilised in the resistant genotype and identify the core regulatory modules that constitute its enhanced immune response.

The identification of a major quantitative trait locus, *qFER4*, provides a genetic anchor for resistance; however, pinpointing the precise causative gene within the interval is essential for mechanistic insight and breeding application [34,35]. To prioritise candidates, we adopted an integrated strategy that combined genomic localisation with dynamic transcriptomic responses to infection [17]. This multi-omics approach enabled us to move beyond positional mapping to evaluate which genes within *qFER4* are both differentially regulated during pathogen attack and carry functionally relevant sequence polymorphisms between resistant and susceptible lines.

By integrating functional annotation of genes within the *qFER4* interval with transcriptomic data at 36 and 72 hpi, we identified four DEGs from the 36 genes in this interval as candidate genes. The expression patterns of these four genes at both time points suggested their potential involvement in the transcriptional response to *F. verticillioides* infection. RT-qPCR validation confirmed that the expression trends were highly consistent with the RNA-seq data, demonstrating the reliability of the transcriptomic analysis. Among these candidates, *Zm00001d053393*, which encodes a Core-2/I-branching β-1,6-N-acetylglucosaminyltransferase, exhibited a consistently downregulated expression pattern following *F. verticillioides* inoculation: its expression levels in the resistant line 3IBZ2 were significantly lower than those in the susceptible line KW5G321 at both 36 and 72 hpi, suggesting a potential role in resistance-associated transcriptional reprogramming. Sequence analysis revealed that the other three candidate genes (*Zm00001d053365*, *Zm00001d053366*, and *Zm00001d053378*) had identical coding sequences between the resistant and susceptible lines, with no amino acid changes detected. Polymorphisms in these genes were confined to promoter regions, including single-nucleotide polymorphisms (SNPs) and insertions/deletions (Indels), which may affect transcriptional regulation but do not alter protein function. In striking contrast, *Zm00001d053393* exhibited extensive structural variations: multiple SNPs and Indels were identified in its promoter region, and more importantly, several SNPs and a single-base insertion were detected in its first and second exons. These variations collectively introduce a premature stop codon in the resistant line 3IBZ2, which is predicted to result in loss of protein function or production of a truncated protein.

Although the biological function of Core-2/I-branching glycosyltransferases in plants remains unclear, studies in mammalian systems have demonstrated their involvement in cell–cell interactions, glycoprotein modification, and immune-related processes [36,37]. Considering the high expression of this gene in the susceptible line and its structural variation-induced functional impairment in the resistant line, we hypothesise that *Zm00001d053393* may function as a susceptibility (S) gene. In susceptible plants, glycan remodelling mediated by functional *Zm00001d053393* may facilitate pathogen attachment or colonisation [38,39], whereas in the resistant line 3IBZ2, loss-of-function mutations and suppressed expression of this gene lead to reduced glycan remodelling, thereby restricting pathogen infection. The candidate gene identified in this study differs functionally from previously reported resistance genes. Cloned resistance genes such as *ZmAuxRP1*, *ZmWAX2*, and *ZmXYXT2* are primarily limited to single aspects of physical barriers or hormone signaling, and the molecular networks they mediate, as well as the breeding potential of their elite haplotypes, require further in-depth investigation [40].

Nonetheless, while the current evidence positions *Zm00001d053393* as the most promising candidate gene within the *qFER4* interval, these findings remain correlational and require further functional validation. To unequivocally establish its role in resistance to maize ear rot, we are currently conducting targeted mutagenesis via CRISPR/Cas9 to knockout *Zm00001d053393* in a susceptible background. Concurrently, we are performing haplotype analysis to identify elite alleles associated with enhanced resistance. The integration of reverse genetic validation with natural variation dissection will not only confirm the function of *Zm00001d053393* but also lay a theoretical foundation for its application in marker-assisted selection and genetic improvement of FER resistance in maize breeding programs.

## 4. Materials and Methods

### 4.1. Plant Materials

The maize inbred lines 3IBZ2 and KW5G321 were obtained from the Institute of Crop Sciences, Chinese Academy of Agricultural Sciences (Beijing, China). Multi-year and multi-location evaluations consistently showed that 3IBZ2 exhibits high resistance to FER, whereas KW5G321 is highly susceptible. These two inbred lines were used as parental materials to construct the segregating population for QTL mapping and subsequent analyses.

### 4.2. Field Inoculation and Disease Assessment

Preparation of Mung Bean Broth: Mung bean broth was prepared by boiling 40 g of mung beans in 1 L of distilled water for approximately 30 min. The mixture was filtered through eight layers of gauze, after which 20 g of sucrose was added and dissolved. The volume was adjusted to 1 L with distilled water, and the broth was sterilised by autoclaving at 121 °C for 30 min. After cooling, fungal mycelial plugs were introduced into the broth, which was incubated at 25 °C with shaking at 160 rpm until abundant conidia were produced. Spore Suspension Preparation: One day before inoculation, the mother culture was diluted with sterile distilled water to a final concentration of 1 × 10^6^ spores/mL, supplemented with 2 μL of Tween 20 per mL to improve spore dispersion. The suspension was mixed thoroughly and stored at 4 °C until inoculation. Field Inoculation: For each maize line, the dates of silking, anthesis, and pollen shedding were recorded. Approximately one week after silk emergence, 2 mL of the spore suspension was injected into the silk channel of each ear using a syringe, with the needle tip positioned at the tip of the ear. Following the injection, the silks were gently pressed to ensure uniform distribution of the suspension. Disease Assessment: At approximately 45 days post-inoculation, husks were manually removed, and ear rot symptoms were recorded. Both disease incidence and severity were assessed using established criteria [41]. The DSI was calculated as:

DSI (%) = ∑ (disease rating score × number of plants at each score)/(maximum disease rating score × total number of plants rated in the line) × 100.

### 4.3. Detection of Reactive Oxygen Species (ROS)

ROS accumulation in maize kernels was assessed using a commercial Plant ROS Detection Kit (NBT method; Solarbio, Beijing, China; Cat. No. G4816). Kernels harvested at 25 days after pollination at 0 and 48 hpi from 3IBZ2 and KW5G321 were immediately immersed in NBT staining solution and incubated overnight at room temperature in the dark. After blue-green formazan precipitates became clearly visible, kernels were rinsed with distilled water and destained in absolute ethanol. The stained kernels were photographed for documentation and comparative analysis.

### 4.4. Determination of FUM Content

Five FER-inoculated ears per maize line were harvested, manually threshed, and thoroughly mixed. Approximately 300 g of kernels were ground using an automated grinder (Beijing HED Technology Co., Ltd., Beijing, China; N1324), sieved through a 20-mesh screen, and stored at 4 °C until analysis. FUM concentration was quantified using a commercial ELISA kit (Biorigin, Beijing, China; Cat. No. BN53455), which employs competitive enzyme-linked immunosorbent detection. According to the manufacturer’s protocol, ground samples were prepared with methanol and water, filtered, and then analysed by ELISA-based quantification.

### 4.5. Construction of Resistant and Susceptible Gene Pools

The resistant inbred line 3IBZ2 (referred to as AMD242 in the initial BSA analysis) was used as the paternal parent, and the susceptible inbred line KW5G321 served as the maternal parent to generate F_1_ hybrids. The F_1_ plants were self-pollinated to produce an F_2_ population. In 2024, the parents and 739 F_2_ individuals were planted in Sanya, Hainan Province (18.27° N, 108.57° E) under standard field management practices. Each plot consisted of a 4 m row containing approximately 17 plants, with a row spacing of 0.6 m. At the seedling stage, each F_2_ individual and the two parental lines were labelled, and approximately 4 cm^2^ of young leaf tissue was collected, immediately flash-frozen in liquid nitrogen, and stored at −80 °C until DNA extraction. Based on disease severity scores following artificial inoculation, 30 highly resistant individuals (score = 1) and 30 highly susceptible individuals (score = 9) were selected to construct the extreme DNA bulks. Genomic DNA was extracted using the cetyltrimethylammonium bromide (CTAB) method, quantified, and pooled in equimolar amounts to generate the resistant and susceptible bulks [42].

### 4.6. Bulked Segregant Analysis Coupled with Sequencing

BSA-seq was used to identify the genes regulating FER in the F_2_ population. We selected 30 plants that were extremely resistant and susceptible to FER to create an extreme population. Genomic DNA from the parental lines and the two bulked DNA pools was subjected to quality assessment before library construction. Four sequencing libraries (two parental and two bulked) were prepared and sequenced on the Illumina HiSeq platform (Illumina, Inc., San Diego, CA, USA), generating an average sequencing depth of 30× for each pool and 10× for each parental line. Library preparation, sequencing, and primary bioinformatic analyses were performed by Novogene Bioinformatics Technology Co., Ltd. (Beijing, China). Raw reads were filtered to remove adapter sequences and low-quality reads, yielding high-quality Clean Reads. Clean Reads were aligned to the maize B73 reference genome (v4) using BWA-backtrack. Duplicate reads were removed using Picard v2, and single-nucleotide polymorphisms (SNPs) were identified using GATK v4. High-confidence SNPs polymorphic between the parents were retained for downstream analyses. Candidate QTL regions were detected using two complementary approaches: Δ(SNP-index) analysis [27] and Euclidean distance (ED) analysis [28]. QTL intervals consistently identified by both methods were considered reliable candidate regions associated with FER resistance. The above work was performed by Novogene Co., Ltd. (Beijing, China).

### 4.7. Genetic Analysis of qFER4 for FER

To determine the inheritance pattern of the major-effect QTL *qFER4*, two polymorphic InDel markers (InDel-1 and InDel-2) were developed within its confidence interval based on sequence polymorphisms between 3IBZ2 and KW5G321. These markers were used to genotype all 739 F_2_ individuals, which were subsequently classified into three genotypic groups: Homozygous resistant (3IBZ2/3IBZ2), Heterozygous (3IBZ2/KW5G321), and Homozygous susceptible (KW5G321/KW5G321). All F_2_ individuals were artificially inoculated with *F. verticillioides*, and disease severity was assessed using a standard 1–9 rating scale. Genotype-phenotype associations were then analysed to characterise the segregation pattern and estimate the genetic effects of *qFER4*.

### 4.8. RNA-Sequencing (RNA-Seq) Analysis

For pathological evaluation, ears of the resistant inbred line 3IBZ2 and the susceptible line KW5G321 were inoculated at 15 days after pollination (DAP) with 2 mL of a *F. verticillioides* conidial suspension (1 × 10^6^ conidia/mL, strain [FvHB202215]), which was injected into the silk channel using a syringe after carefully pulling back the husks, while control ears received sterile 0.01% Tween 20 solution; following inoculation, the husks were repositioned and sealed with parafilm to prevent contamination. Kernels were subsequently collected from a standardized position (the middle of each ear) at 0, 36, and 72 hpi, with three biological replicates per time point—each replicate consisting of kernels pooled from three individual ears—and all samples were immediately frozen in liquid nitrogen and stored at −80 °C for subsequent RNA extraction and transcriptomic analysis. Total RNA was extracted using the TRIzol reagent (Invitrogen, Carlsbad, CA, USA) following the manufacturer’s protocol and submitted to Wuhan Maiwei Metabolic Biotechnology Co., Ltd. for library construction. Sequencing was performed on the Illumina HiSeq™ 2000 platform. Raw reads were processed to remove adapters and low-quality sequences, generating high-quality clean reads. Clean reads were aligned to the maize reference genome (B73_RefGen_v4) using HISAT2 [43]. Gene expression levels were quantified using FPKM values. Differentially expressed genes (DEGs) were identified with thresholds of FDR ≤ 0.01 and |log_2_(fold change)| ≥ 2. GO enrichment analysis was performed to annotate biological processes associated with DEGs, and KEGG pathway enrichment was conducted to identify significantly enriched metabolic and signalling pathways [44].

### 4.9. Quantitative Real-Time PCR (RT-qPCR) Validation

First-strand cDNA was synthesised using the PrimeScript™ 1st Strand cDNA Synthesis Kit (Takara, Japan) according to the manufacturer’s instructions. The maize housekeeping gene ZmUbi served as the internal reference. Primer sequences are provided in Supplementary Table S1. Each 10 μL RT-qPCR reaction contained: 5.0 μL of 2 × Taq Pro Universal SYBR qPCR Master Mix, 0.2 μL each of forward and reverse primers, 2.2 μL of cDNA template, and 2.4 μL of nuclease-free water. Relative gene expression levels were calculated using the 2^−ΔΔCt^ method. Log_2_FC values derived from RT-qPCR were compared with those obtained from RNA-Seq for validation of gene expression patterns [45,46].

### 4.10. Genomic Sequence Alignment

Primers were designed based on genomic and promoter sequences retrieved from the NCBI (https://www.ncbi.nlm.nih.gov/ (accessed on 6 August 2025)) and Gramene (http://www.gramene.org/ (accessed on 6 August 2025)) databases. The promoter regions (2.0 kb upstream of the ATG start codon) and genomic sequences of candidate genes were used as templates for primer design (Supplementary Table S1). Genomic DNA from 3IBZ2 and KW5G321 was used to amplify promoter and genomic fragments of the candidate genes. The coding sequence (CDS) primers were designed based on the cDNA sequences of candidate genes, and their corresponding CDS fragments were amplified from the cDNA of 3IBZ2 and KW5G321. All amplified sequences were aligned using DNAMAN version 7 software to identify SNPs, insertions, deletions, and other structural variations.

### 4.11. Statistical Analysis

Differences in FER resistance between the parental lines and the F_2_ population were analysed using IBM SPSS Statistics 26. Descriptive statistics, including mean, standard deviation, skewness, and kurtosis, were calculated for disease resistance traits. Graphical visualisations were generated using GraphPad Prism 8.

## 5. Conclusions

In this study, one major-effect QTL *qFER4* associated with resistance to *F. verticillioides* ear rot were identified in maize through the BSA-Seq. Transcriptome analysis revealed that the resistant inbred line 3IBZ2 establishes a multilayered defense network, involving phenylpropanoid biosynthesis, JA signaling, MAPK cascades, and plant-pathogen interaction pathways. Within the *qFER4* interval, four candidate genes were further evaluated, among which *Zm00001d053393* displayed substantial structural variations in both its promoter and coding regions. The premature termination stop codon suggests a potential gain of function mutation, positioning this gene as the leading candidate underlying the locus. Genetic characterization analysis indicated that *qFER4* confers resistance in a partially dominant manner. Together, these results provide new genetic resources and mechanistic insights into maize resistance to FER, offering a concrete molecular basis for marker-assisted selection and the breeding of disease-resistant maize varieties.

## Figures and Tables

**Table 1:** Phenotypic variation in parents and F_2_ groups.

	Parents		F_2_						
Trait	3IBZ2	KW5G321	Mean	Maximum	Minimum	SD	Variance	Skewness	Kurtosis
Value	2.26	7.89	4.99	9	1	2.026	4.106	0.269	−0.236

**Table 2:** QTLs conferring effective maize ear rot by 2 methods for identification using BSA-seq.

Method	QTLname	Chr	Pos	Overlapping Value (Mb)	ED Value
Δ(InDel-index)	*qFER4*	4	227.4–230.1	2.7	1.32–1.41
	*qFER6*	6	30.5–31.7	1.2	0.66–1.41
	*qFER7*	7	84.2–85.5	1.3	0.94–1.41
	*qFER8.1*	8	93.2–94.9	1.7	0.64–0.91
	*qFER10.1*	10	32.4–36.3	3.9	0.66–1.41
	*qFER10.2*	10	55.5–59.5	4.0	0.65–1.41
	*qFER10.3*	10	94.4–98.0	3.6	0.81–1.41
Δ(SNP-index)	*qFER8.2*	8	79.6–88.7	9.1	0.63–0.87
	*qFER8.3*	8	113.8–116.7	2.9	0.64–0.86
	*qFER8.4*	8	126.3–131.7	5.4	0.63–0.91
	*qFER9*	9	156.9–158.9	2.0	0.63–0.88
	*qFER10.4*	10	103.7–105.5	1.8	0.65–0.66
	*qFER10.5*	10	113.3–117.8	4.5	0.64–0.82

## Data Availability

Data are available from the authors upon request.

## Supplementary Materials

The following supporting information can be downloaded at: https://www.mdpi.com/article/10.3390/plants15060985/s1, Figure S1: sequence analysis of promoter, CDS and protein in KW5G321 and 3IBZ2; Table S1: List of primer sequences; Table S2: Statistics of SNP Detection and Annotation Results; Table S3: Statistics of InDel Detection and Annotation Results; Table S4: DEGs within the interval and expression level at 36hpi and 72hpi.
