# Supplementary material for: Integrating BSA-Seq and RNA-Seq to Identify Major QTLs and Candidate Genes Conferring Resistance to Fusarium Ear Rot in Maize
Source: Plants (Basel). 2026 Mar 23;15(6):985. doi: 10.3390/plants15060985 (PMC13030315; doi:10.3390/plants15060985)
Supplement: Supplementary file 1 [file plants-15-00985-s001.zip › Supplementary Figure.pdf]

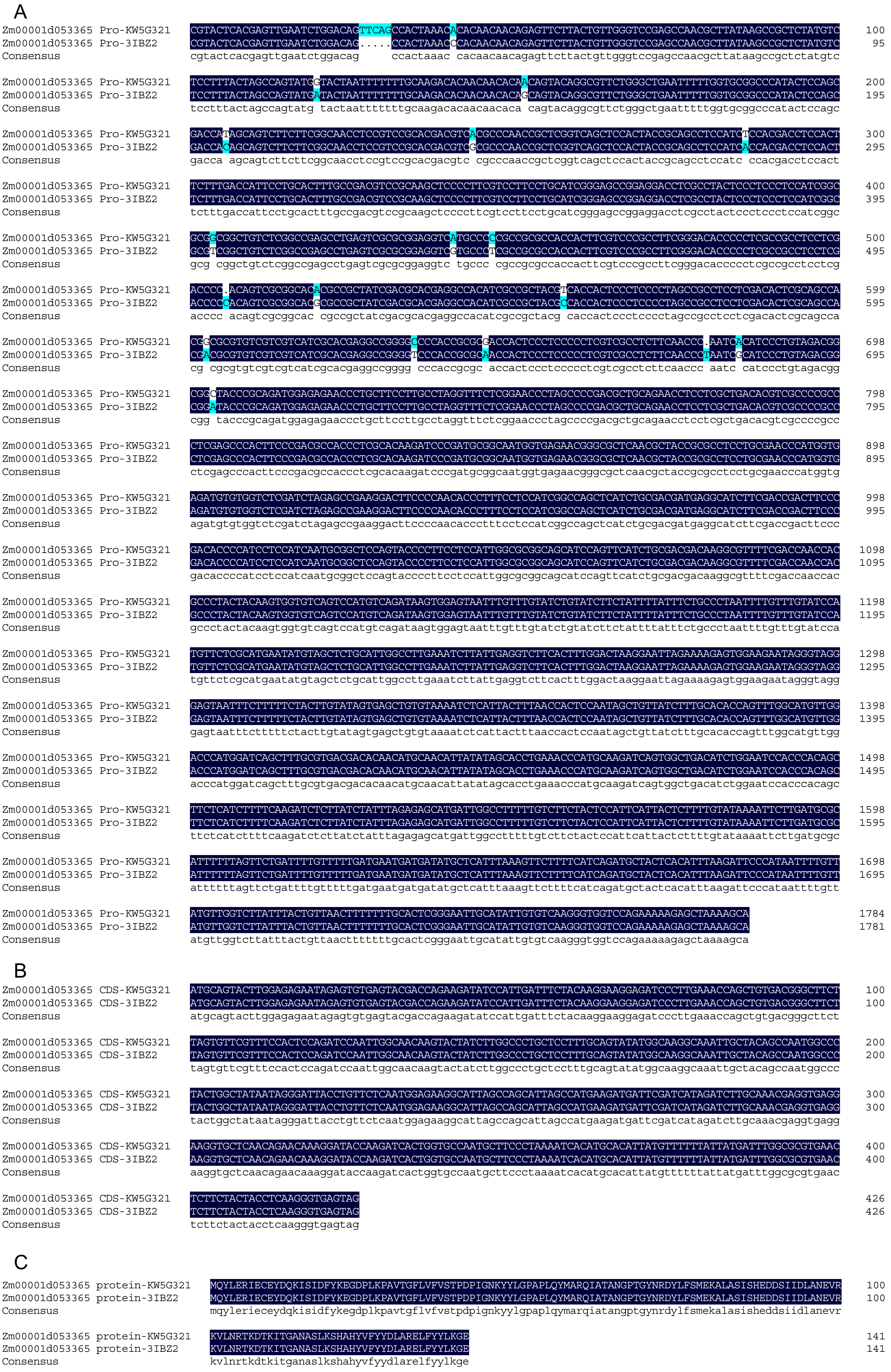
A
Zm00001d053365 Pro-KW5G321
Zm00001d053365 Pro-3IBZ2
Consensus
1784
1781
B
Zm00001d053365 CDS-KW5G321
Zm00001d053365 CDS-3IBZ2
Consensus
426
C
Zm00001d053365 protein-KW5G321
Zm00001d053365 protein-3IBZ2
Consensus
141

D

```
Zm00001d053366 Pro-KW5G321   TGTGTACTTTAGAAGGGAA.TAAATCAAGAAGCACTGCTAACTAGAAGTTCTGTTTAAATTTTTCTGAAGCGGAAAAACTCAGGGAGAAGCTGAGGCAGG    99
Zm00001d053366 Pro-3IBZ2     TGTGTACTTTAGAAGGGAAATAAATCAAGAAGCACTGCTAACTAGAAGTTCTGTTTAAATTTTTCTGAAGCGGAAAAACTCAGGGAGAAGCTGAGGCAGG   100
Consensus                    tgtgtactttagaagggaa taaatcaagaagcactgctaactagaagttctgtttaaatttttctgaagcggaaaaactcagggagaagctgaggcagg

Zm00001d053366 Pro-KW5G321   AGTGCCTGCGGAAGGGGACAAAACTGGCGCATGGGGTGGCTGACGCCCTCTTCGCTGTACCACACACCGAGGATAAAGGCATTGAAGAGCACTAAGGGGA   199
Zm00001d053366 Pro-3IBZ2     AGTGCCTGCGGAAGGGGACAAAACTGGCGCATGGGGTGGCTGACGCCCTCTTCGCTGTACCACACACCGAGGATAAAGGCATTGAAGAGCACTAAGGGGA   200
Consensus                    agtgcctgcggaaggggacaaaactggcgcatggggtggctgacgccctcttcgctgtaccacacaccgaggataaaggcattgaagagcactaagggga

Zm00001d053366 Pro-KW5G321   CATGTCTAGCCTATATTGAGCGCTACGCTACATAAACATATCTTCTATCTATAATTTATTTACCTTTCCTATAAATAAGTTTTTTTATTTATTTCAGCTT   299
Zm00001d053366 Pro-3IBZ2     CATGTCTAGCCTATATTGAGCGCTACGCTACATAAACATATCTTCTATCTATAATTTATTTACCTTTCCTATAAATAAGTTTTTTTATTTATTTCAGCTT   300
Consensus                    catgtctagcctatattgagcgctacgctacataaacatatcttctatctataatttatttacctttcctataaataagtttttttatttatttcagctt

Zm00001d053366 Pro-KW5G321   GCTCATCCTCATGTAGCTCCAATCCATCTGTCATAGGTCTCGAGCAAGCAACTTCGTGATGATTTGATGACCATGCTCATTGCTTGGTCATAAGACTTCT   399
Zm00001d053366 Pro-3IBZ2     GCTCATCCTCATGTAGCTCCAATCCATCTGTCATAGGTCTCGAGCAAGCAACTTCGTGATGATTTGATGACCATGCTCATTGCTTGGTCATAAGACTTCT   400
Consensus                    gctcatcctcatgtagctccaatccatctgtcataggtctcgagcaagcaacttcgtgatgatttgatgaccatgctcattgcttggtcataagacttct

Zm00001d053366 Pro-KW5G321   GTAGCAATCTTGGCATGGACATTTTATCTTCTATTCAAAGCATGCTATATTAGTATCCAAAGCTCGCTCTGTAGTAACTTATAATATCCAATGCAGTGGT   499
Zm00001d053366 Pro-3IBZ2     GTAGCAATCTTGGCATGGACATTTTATCTTCTATCCAAAGCATGCTATATTAGTATCCAAAGCTCGCTCTGTAGTAACTTATAATATCCAATGCAGTGGT   500
Consensus                    gtagcaatcttggcatggacattttatcttctat caaagcatgctatattagtatccaaagctcgctctgtagtaacttataatatccaatgcagtggt

Zm00001d053366 Pro-KW5G321   GTTTTTTATATTGTCTTTGTAACTTACATGTAGCTTAATATTTGTGTATTTGATTTGATCTCACCATCTTATAATAGATGATGATGAGAAACCTCAGCAT   599
Zm00001d053366 Pro-3IBZ2     GTTTTTTATATTGTCTTTGTAACTTACATGTAGCTTAATATTTGTGTATTTGATTTGATCTCACCATCTTATAATAGATGATGATGAGAAACCTCAGCAT   600
Consensus                    gttttttatattgtctttgtaacttacatgtagcttaatatttgtgtatttgatttgatctcaccatcttataatagatgatgatgagaaacctcagcat

Zm00001d053366 Pro-KW5G321   ATATACACACTTCCTAGATTAATATTGAGTTTTATGGTTAGTTGTAGTAACCTTTTAAACCATAGTTCCTAGACTTATCTTGATCAATACTTGTAACTTA   699
Zm00001d053366 Pro-3IBZ2     ATATACACACTTCCTAGATTAATATTGAGTTTTATGGTTAGTTGTAGTAACCTTTTAAACCATAGTTCCTAGACTTATCTTGATCAATACTTGTAACTTA   700
Consensus                    atatacacacttcctagattaatattgagttttatggttagttgtagtaaccttttaaaccatagttcctagacttatcttgatcaatacttgtaactta

Zm00001d053366 Pro-KW5G321   TAATATCCAATGCAGTGGTGTTTTTTCATTTTCAGTGTTTCCATTTTCTCTCGCTACTGCTTCAGCCACCTTTCCTACCCATCTCATACCAACTTTACAT   799
Zm00001d053366 Pro-3IBZ2     TAATATCCAATGCAGTGGTGTTTTTTCATTTTCAGTGTTTCCATTTTCTCTCGCTACTGCTTCAGCCACCTTTCCTACCCATCTCATACCAACTTTACAT   800
Consensus                    taatatccaatgcagtggtgttttttcattttcagtgtttccattttctctcgctactgcttcagccacctttcctacccatctcataccaactttacat

Zm00001d053366 Pro-KW5G321   TACTACAATTTTTTCTTCATCAAATCCTTTTTGGGAGGCTTGGAAAGGTAATTTAGTACATAAATGTTGTGCTTTTAAAATGATTGATGCATATTTCTGT   899
Zm00001d053366 Pro-3IBZ2     TACTACAATTTTTTCTTCATCAAATCCTTTTTGGGAGGCTTGGAAAGGTAATTTAGTACATAAATGTTGTGCTTTTAAAATGATTGATGCATATTTCTGT   900
Consensus                    tactacaattttttcttcatcaaatcctttttgggaggcttggaaaggtaatttagtacataaatgttgtgcttttaaaatgattgatgcatatttctgt

Zm00001d053366 Pro-KW5G321   TGTGGTTTTTTTCTAGACTTCAATTGACCATTTTATATGAGTCATTTAAAAGCATAGTAAAAGCTTCCATAGGTTTAATCCTATGAAACAATCTATTTTA   999
Zm00001d053366 Pro-3IBZ2     TGTGGTTTTTTTCTAGACTTCAATTGACCATTTTATATGAGTCATTTAAAAGCATAGTAAAAGCTTCCATAGGTTTAATCCTATGAAACAATCTATTTTA  1000
Consensus                    tgtggtttttttctagacttcaattgaccattttatatgagtcatttaaaagcatagtaaaagcttccataggtttaatcctatgaaacaatctatttta

Zm00001d053366 Pro-KW5G321   GACTAGCATCTTACTTCCTTTGATAATTAAAGAAGGGACAAAAAAATCCTGCTCATCTCATCCTTAAGTTTGATGTTTGCTATTAGTGTTGAACATAGTT  1099
Zm00001d053366 Pro-3IBZ2     GACTAGCATCTTACTTCCTTTGATAATTAAAGAAGGGACAAAAAAATCCTGCTCATCTCATCCTTAAGTTTGATGTTTGCTATTAGTGTTGAACATAGTT  1100
Consensus                    gactagcatcttacttcctttgataattaaagaagggacaaaaaaatcctgctcatctcatccttaagtttgatgtttgctattagtgttgaacatagtt

Zm00001d053366 Pro-KW5G321   CTACTATGCAAGTAATTCACCTACATTGCACTCCTTTATGCCCATTTTTGGGAACACTAGGATGGTTATTGAGAATAGTCTAGAACAAAGGACACTTCAA  1199
Zm00001d053366 Pro-3IBZ2     CTACTATGCAAGTAATTCACCTACATTGCACTCCTTTATGCCCATTTTTGGGAACACTAGGATGGTTATTGAGAATAGTCTAGAACAAAGGACACTTCAA  1200
Consensus                    ctactatgcaagtaattcacctacattgcactcctttatgcccatttttgggaacactaggatggttattgagaatagtctagaacaaaggacacttcaa

Zm00001d053366 Pro-KW5G321   TTTTGAATGTATGTGTCAATTTTGAATCTCCGTTTGCAGGGAAGGGTGCTCCCAGCCCCCGTCGTGCCCCTCGGCCCCTCCCAACCCTCGTCGTCGTAAC  1299
Zm00001d053366 Pro-3IBZ2     TTTTGAATGTATGTGTCAATTTTGAATCTCCGTTTGCAGGGAAGGGTGCTCCCAGCCCCCGTCGTGCCCCTCGGCCCCTCCCAACCCTCGTCGTCGTAAC  1300
Consensus                    ttttgaatgtatgtgtcaattttgaatctccgtttgcagggaagggtgctcccagcccccgtcgtgcccctcggcccctcccaaccctcgtcgtcgtaac

Zm00001d053366 Pro-KW5G321   GGAGGTATATTCTGCCCTGAATAAAATGATTTCTTCTGTATTGTACTTACACGTGTTTTTATAAGGATGTAAGGAGGGCCCAAAACCTCGCCCCAAATCG  1399
Zm00001d053366 Pro-3IBZ2     GGAGGTATATTCTGCCCTGAATAAAATGATTTCTTCTGTATTGTACTTACACGTGTTTTTATAAGGATGTAAGGAGGGCCCAAAACCTCGCCCCAAATCG  1400
Consensus                    ggaggtatattctgccctgaataaaatgatttcttctgtattgtacttacacgtgtttttataaggatgtaaggagggcccaaaacctcgccccaaatcg

Zm00001d053366 Pro-KW5G321   ACGCGCATTTGGGTGTTCGCTTAATCGTCCTATTTAAATCAGCAGGGTGTTGCGACAAGCCAGCAGATGAGCGTGGCGATCCCGCTGGAGCAGTCCATCA  1499
Zm00001d053366 Pro-3IBZ2     ACGCGCATTTGGGTGTTCGCTTAATCGTCCTATTTAAATCAGCAGGGTGTTGCGACAAGCCAGCAGATGAGCGTGGCGATCCCGCTGGAGCAGTCCATCA  1500
Consensus                    acgcgcatttgggtgttcgcttaatcgtcctatttaaatcagcagggtgttgcgacaagccagcagatgagcgtggcgatcccgctggagcagtccatca

Zm00001d053366 Pro-KW5G321   CGCCGGTGCTTCAGGACTTTATGAATCTCAACATATTCTATCTACCAGTGTATTACTGTGGAGGTGAGGAGTATAGCTCAAAGTATGATCTGCTCCTCTT  1599
Zm00001d053366 Pro-3IBZ2     CGCCGGTGCTTCAGGACTTTATGAATCTCAACATATTCTATCTACCAGTGTATTACTGTGGAGGTGAGGAGTATAGCTCAAAGTATGATCTGCTCCTCTT  1600
Consensus                    cgccggtgcttcaggactttatgaatctcaacatattctatctaccagtgtattactgtggaggtgaggagtatagctcaaagtatgatctgctcctctt

Zm00001d053366 Pro-KW5G321   CTCTGCTATCCTTCTCCGCTCCCCTCATGCTCCACCATCACCGTCGACATGGAAACTAGAGTTGTCCCTCCACTAGCCGCATGGATCCTCTCTGCCTTTA  1699
Zm00001d053366 Pro-3IBZ2     CTCTGCTATCCTTCTCCGCTCCCCTCATGCTCCACCATCACCGTCGACATGGAAACTAGAGTTGTCCCTCCACTAGCCGCATGGATCCTCTCTGCCTTTA  1700
Consensus                    ctctgctatccttctccgctcccctcatgctccaccatcaccgtcgacatggaaactagagttgtccctccactagccgcatggatcctctctgccttta

Zm00001d053366 Pro-KW5G321   GTTCCTCGCCTCTTCTCTCTCTTTGCAGGTCTGCGAGGTAGTACCATCGACGAGGCTTCTCCATCCCCTCCGGCGAGCTCCCCTTCCAAAAAAAGTGGTG  1799
Zm00001d053366 Pro-3IBZ2     GTTCCTCGCCTCTTCTCTCTCTTTGCAGGTCTGCGAGGTAGTACCATCGACGAGGCTTCTCCATCCCCTCCGGCGAGCTCCCCTTCCAAAAAAAGTGGTG  1800
Consensus                    gttcctcgcctcttctctctctttgcaggtctgcgaggtagtaccatcgacgaggcttctccatcccctccggcgagctccccttccaaaaaaagtggtg

Zm00001d053366 Pro-KW5G321   AGGTATGCCTGCCCTCATTCCTTACCTCTCGTACATGCCCTTAGTACTAATATCGCCATAGATGGTGCATACAAAACGAAACTGCTTTTGTTGACCCCGT  1899
Zm00001d053366 Pro-3IBZ2     AGGTATGCCTGCCCTCATTCCTTACCTCTCGTACATGCCCTTAGTACTAATATCGCCATAGATGGTGCATACAAAACGAAACTGCTTTTGT.GACCCCGT  1899
Consensus                    aggtatgcctgccctcattccttacctctcgtacatgcccttagtactaatatcgccatagatggtgcatacaaaacgaaactgcttttgt gaccccgt

Zm00001d053366 Pro-KW5G321   GCAGCAGGATTATTGCTTAACA                                                                                1921
Zm00001d053366 Pro-3IBZ2     GCAGCAGGATTATTGCTTAACA                                                                                1921
Consensus                    gcagcaggattattgcttaaca
```

E

```
Zm00001d053366 CDS-KW5G321   ATGGCGGTGAATGAAGGAATGAACGAAATTGACGTGTGGTCTGTGGGCTGTATCTTTGCGGAAATGGTGAACCAAAAGCCACTATTCCCTGGTGATTCTG   100
Zm00001d053366 CDS-3IBZ2     ATGGCGGTGAATGAAGGAATGAACGAAATTGACGTGTGGTCTGTGGGCTGTATCTTTGCGGAAATGGTGAACCAAAAGCCACTATTCCCTGGTGATTCTG   100
Consensus                    atggcggtgaatgaaggaatgaacgaaattgacgtgtggtctgtgggctgtatctttgcggaaatggtgaaccaaaagccactattccctggtgattctg

Zm00001d053366 CDS-KW5G321   AGATCGACGAACTGTTCAAGATATTCAGGGTGCTAGGTACACCGAATGAACAAAGTTGGCCTGGAGTCAGTTGTTTGCCTGACTTCAAGACTGCTTTCCC   200
Zm00001d053366 CDS-3IBZ2     AGATCGACGAACTGTTCAAGATATTCAGGGTGCTAGGTACACCGAATGAACAAAGTTGGCCTGGAGTCAGTTGTTTGCCTGACTTCAAGACTGCTTTCCC   200
Consensus                    agatcgacgaactgttcaagatattcagggtgctaggtacaccgaatgaacaaagttggcctggagtcagttgtttgcctgacttcaagactgctttccc

Zm00001d053366 CDS-KW5G321   CAGGTGGCAATCTCAGGACCTGGCAACAATAGTCCCAAATCTTGAACCTGCTGGCTTGGACCTTCTCTCTAAAATGCTTTGA                    282
Zm00001d053366 CDS-3IBZ2     CAGGTGGCAATCTCAGGACCTGGCAACAATAGTCCCAAATCTTGAACCTGCTGGCTTGGACCTTCTCTCTAAAATGCTTTGA                    282
Consensus                    caggtggcaatctcaggacctggcaacaatagtcccaaatcttgaacctgctggcttggaccttctctctaaaatgctttga
```

F

```
Zm00001d053366 protein-KW5G321   MAVNEGMNEIDVWSVGCIFAEMVNQKPLFPGDSEIDELFKIFRVLGTPNEQSWPGVSCLPDFKTAFPRWQSQDLATIVPNLEPAGLDLLSKML   93
Zm00001d053366 protein-3IBZ2     MAVNEGMNEIDVWSVGCIFAEMVNQKPLFPGDSEIDELFKIFRVLGTPNEQSWPGVSCLPDFKTAFPRWQSQDLATIVPNLEPAGLDLLSKML   93
Consensus                        mavnegmneidvwsvgcifaemvnqkplfpgdseidelfkifrvlgtpneqswpgvsclpdfktafprwqsqdlativpnlepagldllskml
```

G

```
Zm00001d053378 Pro-KW5G321   GAGATGAACCTAGAATATTCTTGTCCATATTGATATGGTCTGACTCTAAAAAAAAATCAACACAGCCCTATAGACGCAATGCAGATATATTCTGGTTCTT    100
Zm00001d053378 Pro-3IBZ2     GAGATGAGCCTAGAATATTTTTGTGCATATTGATATGGTGTGACTCTAAAAAAAAATCAACACAGCCCTATAGACGCAATGCAGATATATTCTGGTTCTT    100
Consensus                    gagatga cctagaatatt ttgt catattgatatggt tgactctaaaaaaaaatcaacacagccctatagacgcaatgcagatatattctggttctt

Zm00001d053378 Pro-KW5G321   ACTTTTTGCAGTGATCGAGACATGTTAATATTCTTGTTCCTGATACAAAGCTGCAAGCTGTGGCATGCATGATGAGATTTTGGCTTCTGCGTTCATTTCG    200
Zm00001d053378 Pro-3IBZ2     ACTTTTTGCAGTGATCGAGACATGTTAATATTCTTGTTCCTGATACAAAGCTGCAAGCTGTGGCATGCATGATGAGATTTTGGCTTCTGCGTTCATTTCG    200
Consensus                    actttttgcagtgatcgagacatgttaatattcttgttcctgatacaaagctgcaagctgtggcatgcatgatgagattttggcttctgcgttcatttcg

Zm00001d053378 Pro-KW5G321   CAGGCACATTTGCTGGGAATTAGGGCAATCGACTTTTAAGTACATTTGGTGTACGGCTGTTTGATTATCACTGGTAAGGCTCATAAGCTTCAAATTCTGC    300
Zm00001d053378 Pro-3IBZ2     CAGGCACATTTGCTGGGAATTAGGGCAATCGACTTTTAAGTACATTTGGTGTACGGCTGTTTGATTATCACTGGTAAGGCTCATAAGCTTCAAATTCTGC    300
Consensus                    caggcacatttgctgggaattagggcaatcgacttttaagtacatttggtgtacggctgtttgattatcactggtaaggctcataagcttcaaattctgc

Zm00001d053378 Pro-KW5G321   CACTTTTTATGTTAGAAACTTAGTCCCTACTAGTATTTTCTGTGCAACAGTTTATGGTGCCTGTGGGTTTTATTTTGTCCTTGTCAGGTAGCACTTGGTC    400
Zm00001d053378 Pro-3IBZ2     CACTTTTTATGTTAGAAACTTAGTCCCTACTAGTATTTTCTGTGCAACAGTTTATGGTGCCTGTGGGTTTTATTTTGTCCTTGTCAGGTAGCACTTGGTC    400
Consensus                    cactttttatgttagaaacttagtccctactagtattttctgtgcaacagtttatggtgcctgtgggttttattttgtccttgtcaggtagcacttggtc

Zm00001d053378 Pro-KW5G321   ACTTGAGTTCCTGTAAATTTTTGGAAGTTCAAATTATCTGATCTTAGGTCGAATGGATCAATGATCTAACCATAACTTGCGTCCTCTTGATCTTGTTGGT    500
Zm00001d053378 Pro-3IBZ2     ACTTGAGTTCCTGTAAATTTTTGGAAGTTCAAATTATCTGATCTTAGGTCGAATGGATCAATGATCTAACCATAACTTGCGTCCTCTTGATCTTGTTGGT    500
Consensus                    acttgagttcctgtaaatttttggaagttcaaattatctgatcttaggtcgaatggatcaatgatctaaccataacttgcgtcctcttgatcttgttggt

Zm00001d053378 Pro-KW5G321   GGCCTCTTCTGGACACCGGTGCCTCCTCTCGAACGCTGGTGAGGAAGTAGAGATCAGCGGCCAATGGTGTTGTTTGCAGTGGCCTTCCTGTCACTTGCAG    600
Zm00001d053378 Pro-3IBZ2     GGCCTCTTCTGGACACCGGTGCCTCCTCTCGAACGCTGGTGAGGAAGTAGAGATCAGCGGCCAATGGTGTTGTTTGCAGTGGCCTTCCTGTCACTTGCAG    600
Consensus                    ggcctcttctggacaccggtgcctcctctcgaacgctggtgaggaagtagagatcagcggccaatggtgttgtttgcagtggccttcctgtcacttgcag

Zm00001d053378 Pro-KW5G321   CACCACTTAGATCGTATCCTAGGAAGTTTGTTAGGCTAGCTAATCCCCTATGAACATTGGTTCCCTTGGGTGTGAGCTGTCCCTTTTATTGGCACTGTGT    700
Zm00001d053378 Pro-3IBZ2     CACCACTTAGATCGTATCCTAGGAAGTTTGTTAGGCTAGCTAATCCCCTATGAACATTGGTTCCCTTGGGTGTGAGCTGTCCCTTTTATTGGCACTGTGT    700
Consensus                    caccacttagatcgtatcctaggaagtttgttaggctagctaatcccctatgaacattggttcccttgggtgtgagctgtcccttttattggcactgtgt

Zm00001d053378 Pro-KW5G321   GATGCAGGGCTAACCATCACTCGGTCCAGGGTGCCCTTGGTTGCGGCACATACAGGAGCTACGCACTCTCCTTGACTGGGTTAGAGAAACAGTCTGAGCA    800
Zm00001d053378 Pro-3IBZ2     GATGCAGGGCTAACCATCACTCGGTCCAGGGTGCCCTTGGTTGCGGCACATACAGGAGCTACGCACTCTCCTTGACTGGGTTAGAGAAACAGTCTGAGCA    800
Consensus                    gatgcagggctaaccatcactcggtccagggtgcccttggttgcggcacatacaggagctacgcactctccttgactgggttagagaaacagtctgagca

Zm00001d053378 Pro-KW5G321   TTGGGCCATTCCTATGATCCTATCTCTGAGTCTGAGATCATTTTTCCAACACAAATGAGCAGTGATTCTACTCTTAGCAGTATGGTCTCATATGGAACAA    900
Zm00001d053378 Pro-3IBZ2     TTGGGCCATTCCTATGATCCTATCTCTGAGTCTGAGATCATTTTTCCAACACAAATGAGCAGTGATTCTACTCTTAGCAGTATGGTCTCATATGGAACAA    900
Consensus                    ttgggccattcctatgatcctatctctgagtctgagatcatttttccaacacaaatgagcagtgattctactcttagcagtatggtctcatatggaacaa

Zm00001d053378 Pro-KW5G321   TTTGACATACATTTGGAATTCAGTTGTTTAGCTTTGTGCCTTTCTTTATCATAACATCGTATGCTGAGTTTTTTATTTTCATTTTTAATATAAACCATTT   1000
Zm00001d053378 Pro-3IBZ2     TTTGACATACATTTGGAATTCAGTTGTTTAGCTTTGTGCCTTTCTTTATCATAACATCGTATGCTGAGTTTTTTATTTTCATTTTTAATATAAACCATTT   1000
Consensus                    tttgacatacatttggaattcagttgtttagctttgtgcctttctttatcataacatcgtatgctgagttttttattttcatttttaatataaaccattt

Zm00001d053378 Pro-KW5G321   GAAGGCCATGTTGGGTTGGAACTCACTAAAAGCTCCCAACTATTTAAACAACACTGTGGTAGTTGTCTCTTGCCCTTTTTTCTTGGCATGATTGAACTCC   1100
Zm00001d053378 Pro-3IBZ2     GAAGGCCATGTTGGGTTGGAACTCACTAAAAGCTCCCAACTATTTAAACAACACTGTGGTAGTTGTCTCTTGCCCTTTTTTCTTGGCATGATTGAACTCC   1100
Consensus                    gaaggccatgttgggttggaactcactaaaagctcccaactatttaaacaacactgtggtagttgtctcttgcccttttttcttggcatgattgaactcc

Zm00001d053378 Pro-KW5G321   TCAATCTTCTTAATTCTTTTGGCTATGTAGTATGACTTCCGTCCTGACCTTTTATTGTTGAACTCTGGTTGATCAGTAAGAATGTAAGATTGTGTTTTTG   1200
Zm00001d053378 Pro-3IBZ2     TCAATCTTCTTAATTCTTTTGGCTATGTAGTATGACTTCCGTCCTGACCTTTTATTGTTGAACTCTGGTTGATCAGTAAGAATGTAAGATTGTGTTTTTG   1200
Consensus                    tcaatcttcttaattcttttggctatgtagtatgacttccgtcctgaccttttattgttgaactctggttgatcagtaagaatgtaagattgtgtttttg

Zm00001d053378 Pro-KW5G321   CATACAAGATTTTTCTCTCCCTTTTATTCTTGTCAATGGCATATGTGAAGATTCAACATTGATTACAGATATGGGACTGAATGCACTCTGATACCCAATT   1300
Zm00001d053378 Pro-3IBZ2     CATACAAGATTTTTCTCTCCCTTTTATTCTTGTCAATGGCATATGTGAAGATTCAACATTGATTACAGATATGGGACTGAATGCACTCTGATACCCAATT   1300
Consensus                    catacaagatttttctctcccttttattcttgtcaatggcatatgtgaagattcaacattgattacagatatgggactgaatgcactctgatacccaatt

Zm00001d053378 Pro-KW5G321   GCTTTTCAGCTGTATCCTGTTTATCTAATTGTTGCATATCTGTTACAATCTTTATACATCATTTTCTTTTCTCCATAATTATTTTAACTCAATGAGAACG   1400
Zm00001d053378 Pro-3IBZ2     GCTTTTCAGCTGTATCCTGTTTATCTAATTGTTGCATATCTGTTACAATCTTTATACATCATTTTCTTTTCTCCATAATTATTTTAACTCAATGAGAACG   1400
Consensus                    gcttttcagctgtatcctgtttatctaattgttgcatatctgttacaatctttatacatcattttcttttctccataattattttaactcaatgagaacg

Zm00001d053378 Pro-KW5G321   TATCTCATCTGTATTCTGTTCACTTCCTGCAGAGATTCAGTCATTCGGTTGTATGAGAGTTCTGGCTGGAGACTTACGAGTGTAGCACTGTTGTCGGCTT   1500
Zm00001d053378 Pro-3IBZ2     TATCTCATCTGTATTCTGTTCACTTCCTGCAGAGATTCAGTCATTCGGTTGTATGAGAGTTCTGGCTGGAGACTTACGAGTGTAGCACTGTTGTCGGCTT   1500
Consensus                    tatctcatctgtattctgttcacttcctgcagagattcagtcattcggttgtatgagagttctggctggagacttacgagtgtagcactgttgtcggctt

Zm00001d053378 Pro-KW5G321   GTGATTAAATAGTATCACTTCACATACAGCACTGGATAATGGATCGTAGGGATACTTCAGGGTTTGTTAGTGGAGGGTGCAAAAATAATGAAGCCTACTG   1600
Zm00001d053378 Pro-3IBZ2     GTGATTAAATAGTATCACTTCACATACAGCACTGGATAATGGATCGTAGGGATACTTCAGGGTTTGTTAGTGGAGGGTGCAAAAATAATGAAGCCTACTG   1600
Consensus                    gtgattaaatagtatcacttcacatacagcactggataatggatcgtagggatacttcagggtttgttagtggagggtgcaaaaataatgaagcctactg

Zm00001d053378 Pro-KW5G321   ATTTCTGTAGATCTTCTAAATTGGTAGCTTTGTTGACAATTATTTAACCCAGAAGTGAGGCCAGCTGGTACTTCCCATAAAGCTTGGTTACTCTAGGAGC   1700
Zm00001d053378 Pro-3IBZ2     ATTTCTGTAGATCTTCTAAATTGGTAGCTTTGTTGACAATTATTTAACCCAGAAGTGAGGCCAGCTGGTACTTCCCATAAAGCTTGGTTACTCTAGGAGC   1700
Consensus                    atttctgtagatcttctaaattggtagctttgttgacaattatttaacccagaagtgaggccagctggtacttcccataaagcttggttactctaggagc

Zm00001d053378 Pro-KW5G321   A                                                                                                      1701
Zm00001d053378 Pro-3IBZ2     A                                                                                                      1701
Consensus                    a
```

H

```
Zm00001d053378 CDS-KW5G321   ATGGATCCCAGGCACGGGCGTTCAGCATTCACTATAAGTTTCTCAAATCCAAATGGCTTTGCGTATCAGTGCACTCCCATTCCAGCCGTCAGTTATCCAG   100
Zm00001d053378 CDS-3IBZ2    ATGGATCCCAGGCACGGGCGTTCAGCATTCACTATAAGTTTCTCAAATCCAAATGGCTTTGCGTATCAGTGCACTCCCATTCCAGCCGTCAGTTATCCAG   100
Consensus                    atggatcccaggcacgggcgttcagcattcactataagtttctcaaatccaaatggctttgcgtatcagtgcactcccattccagccgtcagttatccag

Zm00001d053378 CDS-KW5G321   CAACTGGTTCTAGCCAGCTTGATGTGCTAGCTCCTAGAGGTTGCAAGAGAAAGTGGACTGAGTTGGCCCTAGGTCTGGGTGACTCATCAAGCTCAGACAG   200
Zm00001d053378 CDS-3IBZ2    CAACTGGTTCTAGCCAGCTTGATGTGCTAGCTCCTAGAGGTTGCAAGAGAAAGTGGACTGAGTTGGCCCTAGGTCTGGGTGACTCATCAAGCTCAGACAG   200
Consensus                    caactggttctagccagcttgatgtgctagctcctagaggttgcaagagaaagtggactgagttggccctaggtctgggtgactcatcaagctcagacag

Zm00001d053378 CDS-KW5G321   CAGCAAGAGGAGCATGGGTACTGGCTGCACTGTTTCTTCTGCCAAGGGAAGCGATGACGTCTCATGTATGGACTACGACATAGGTTTTGAGCTATCTCTC   300
Zm00001d053378 CDS-3IBZ2    CAGCAAGAGGAGCATGGGTACTGGCTGCACTGTTTCTTCTGCCAAGGGAAGCGATGACGTCTCATGTATGGACTACGACATAGGTTTTGAGCTATCTCTC   300
Consensus                    cagcaagaggagcatgggtactggctgcactgtttcttctgccaagggaagcgatgacgtctcatgtatggactacgacataggttttgagctatctctc

Zm00001d053378 CDS-KW5G321   GGCAATGAAGGTACTTCGAGGCTGTGTAAACAGGCCTGTGACTCTACAAGGACCATGGAGAAGCCTGGGTTGGATCTTAAGTTATCTTTGGCTCCATCTC   400
Zm00001d053378 CDS-3IBZ2    GGCAATGAAGGTACTTCGAGGCTGTGTAAACAGGCCTGTGACTCTACAAGGACCATGGAGAAGCCTGGGTTGGATCTTAAGTTATCTTTGGCTCCATCTC   400
Consensus                    ggcaatgaaggtacttcgaggctgtgtaaacaggcctgtgactctacaaggaccatggagaagcctgggttggatcttaagttatctttggctccatctc

Zm00001d053378 CDS-KW5G321   AATCTGATGTAACTGATGCAGATGTAATTAGAAGCAGTGCACCTCAGGACATGTTTGTGCATCATCAGTACTTAATGTCGTCAGCGCCAACAGTTGATGA   500
Zm00001d053378 CDS-3IBZ2    AATCTGATGTAACTGATGCAGATGTAATTAGAAGCAGTGCACCTCAGGACATGTTTGTGCATCATCAGTACTTAATGTCGTCAGCGCCAACAGTTGATGA   500
Consensus                    aatctgatgtaactgatgcagatgtaattagaagcagtgcacctcaggacatgtttgtgcatcatcagtacttaatgtcgtcagcgccaacagttgatga

Zm00001d053378 CDS-KW5G321   AGGATCTACATCCGCTCGACGCTTATCTGGAGGCATGGTGGGTTCCTTTCTTAACCAGGATGGGATTTCTCTGGACCAAGCGCTCCCGGTTAACGCTAAT   600
Zm00001d053378 CDS-3IBZ2    AGGATCTACATCCGCTCGACGCTTATCTGGAGGCATGGTGGGTTCCTTTCTTAACCAGGATGGGATTTCTCTGGACCAAGCGCTCCCGGTTAACGCTAAT   600
Consensus                    aggatctacatccgctcgacgcttatctggaggcatggtgggttcctttcttaaccaggatgggatttctctggaccaagcgctcccggttaacgctaat

Zm00001d053378 CDS-KW5G321   CAGGTCCAAGGTCCAGCTCCTTCAGCACCAACAGTGCTCCAACTGCCAAAAAGTTCAGCTGCCTCTTCTTCTGGGTTTGTCCATCCACAGCAGCGCAGCA   700
Zm00001d053378 CDS-3IBZ2    CAGGTCCAAGGTCCAGCTCCTTCAGCACCAACAGTGCTCCAACTGCCAAAAAGTTCAGCTGCCTCTTCTTCTGGGTTTGTCCATCCACAGCAGCGCAGCA   700
Consensus                    caggtccaaggtccagctccttcagcaccaacagtgctccaactgccaaaaagttcagctgcctcttcttctgggtttgtccatccacagcagcgcagca

Zm00001d053378 CDS-KW5G321   GTATCACAAAAATCTGTTCACACCCAGGCTGTGCAAAAGGAGCGAGGGGTTCATCTGGGCGGTGCATTGCGCACGGTGGGGGTAGAAGGTGCCAGAAAGG   800
Zm00001d053378 CDS-3IBZ2    GTATCACAAAAATCTGTTCACACCCAGGCTGTGCAAAAGGAGCGAGGGGTTCATCTGGGCGGTGCATTGCGCACGGTGGGGGTAGAAGGTGCCAGAAAGG   800
Consensus                    gtatcacaaaaatctgttcacacccaggctgtgcaaaaggagcgaggggttcatctgggcggtgcattgcgcacggtgggggtagaaggtgccagaaagg

Zm00001d053378 CDS-KW5G321   AGGCTGCAGCAAAGGAGCTGAGGGAAAGACCATCTTCTGTAAAGCCCATGGAGGGGGGAAGCGGTGTGAACACCTTGGATGCACCAAAAGTGCGGAAGGC   900
Zm00001d053378 CDS-3IBZ2    AGGCTGCAGCAAAGGAGCTGAGGGAAAGACCATCTTCTGTAAAGCCCATGGAGGGGGGAAGCGGTGTGAACACCTTGGATGCACCAAAAGTGCGGAAGGC   900
Consensus                    aggctgcagcaaaggagctgagggaaagaccatcttctgtaaagcccatggaggggggaagcggtgtgaacaccttggatgcaccaaaagtgcggaaggc

Zm00001d053378 CDS-KW5G321   CGGACTGATTTCTGCATAGCCCATGGCGGCGGGCGGCGCTGCAGCCATGAAGGGTGCAAGAGGGCAGCACGAGGCAAATCGGGCCTCTGTATCAAGCACG   1000
Zm00001d053378 CDS-3IBZ2    CGGACTGATTTCTGCATAGCCCATGGCGGCGGGCGGCGCTGCAGCCATGAAGGGTGCAAGAGGGCAGCACGAGGCAAATCGGGCCTCTGTATCAAGCACG   1000
Consensus                    cggactgatttctgcatagcccatggcggcgggcggcgctgcagccatgaagggtgcaagagggcagcacgaggcaaatcgggcctctgtatcaagcacg

Zm00001d053378 CDS-KW5G321   GTGGTGGGAAGAGGTGCGAAAAGCCAAACTGCACAAAGAGCGCCGAAGGGCGTTCAGGCATGTGCATTGCCCACGGTGGCGGGCGCCGCTGCCAGTACGC   1100
Zm00001d053378 CDS-3IBZ2    GTGGTGGGAAGAGGTGCGAAAAGCCAAACTGCACAAAGAGCGCCGAAGGGCGTTCAGGCATGTGCATTGCCCACGGTGGCGGGCGCCGCTGCCAGTACGC   1100
Consensus                    gtggtgggaagaggtgcgaaaagccaaactgcacaaagagcgccgaagggcgttcaggcatgtgcattgcccacggtggcgggcgccgctgccagtacgc

Zm00001d053378 CDS-KW5G321   TGGCTGCGGGAAGGGAGCTCAGGGCAGCACCAGTTTCTGCAAAGCCCACGGTGGCGGCAAGAGATGCACACGCCCCGACTGTTCCAAGGGCGCGGAGGGA   1200
Zm00001d053378 CDS-3IBZ2    TGGCTGCGGGAAGGGAGCTCAGGGCAGCACCAGTTTCTGCAAAGCCCACGGTGGCGGCAAGAGATGCACACGCCCCGACTGTTCCAAGGGCGCGGAGGGA   1200
Consensus                    tggctgcgggaagggagctcagggcagcaccagtttctgcaaagcccacggtggcggcaagagatgcacacgccccgactgttccaagggcgcggaggga

Zm00001d053378 CDS-KW5G321   AGCACGGCGTTCTGCAAAGCGCACGGGGGCGGCAAGCGATGCTCGGCTGACGGGTGCACGAAGAGCGTGCATGGTGGGACCCAGTTCTGCGTCGCGCATG   1300
Zm00001d053378 CDS-3IBZ2    AGCACGGCGTTCTGCAAAGCGCACGGGGGCGGCAAGCGATGCTCGGCTGACGGGTGCACGAAGAGCGTGCATGGTGGGACCCAGTTCTGCGTCGCGCATG   1300
Consensus                    agcacggcgttctgcaaagcgcacgggggcggcaagcgatgctcggctgacgggtgcacgaagagcgtgcatggtgggacccagttctgcgtcgcgcatg

Zm00001d053378 CDS-KW5G321   GAGGCGGGAAGAGATGCGTGGTAGAAGGATGCAGGAAGAGCGCGAGAGGCCGGAGCGACCGCTGCGTCGGGCACGGCGGGGGCAAGCGGTGCCACTCCGC   1400
Zm00001d053378 CDS-3IBZ2    GAGGCGGGAAGAGATGCGTGGTAGAAGGATGCAGGAAGAGCGCGAGAGGCCGGAGCGACCGCTGCGTCGGGCACGGCGGGGGCAAGCGGTGCCACTCCGC   1400
Consensus                    gaggcgggaagagatgcgtggtagaaggatgcaggaagagcgcgagaggccggagcgaccgctgcgtcgggcacggcgggggcaagcggtgccactccgc

Zm00001d053378 CDS-KW5G321   TGGCTGTGGGAAGAGCGCACAGGGAAGCACCGATTTCTGCAAGTCGCACGGTGGAGGCAGGCGCTGTTGGTGGGGACATGTCGGGTCGGGTGGCGCTCCT   1500
Zm00001d053378 CDS-3IBZ2    TGGCTGTGGGAAGAGCGCACAGGGAAGCACCGATTTCTGCAAGTCGCACGGTGGAGGCAGGCGCTGTTGGTGGGGACATGTCGGGTCGGGTGGCGCTCCT   1500
Consensus                    tggctgtgggaagagcgcacagggaagcaccgatttctgcaagtcgcacggtggaggcaggcgctgttggtggggacatgtcgggtcgggtggcgctcct

Zm00001d053378 CDS-KW5G321   TGCGACCGTCTGGCGAGAGGCAGAAAGGGGCTATGCGATCGACACAACCCGCTGGTGGACGACAACAGCGTCCACGGGGGTGTATCGTTTGGTGGGTTTT   1600
Zm00001d053378 CDS-3IBZ2    TGCGACCGTCTGGCGAGAGGCAGAAAGGGGCTATGCGATCGACACAACCCGCTGGTGGACGACAACAGCGTCCACGGGGGTGTATCGTTTGGTGGGTTTT   1600
Consensus                    tgcgaccgtctggcgagaggcagaaaggggctatgcgatcgacacaacccgctggtggacgacaacagcgtccacgggggtgtatcgtttggtgggtttt

Zm00001d053378 CDS-KW5G321   CCACCCTTTCTGAGGGAGGCGCCGAAACAAGCTTCTTCATGCACACGGTGGAGGATCCTCGCCACGTGGCGCCATCGGCCCGTGAAGGCCGGGTGCATGG   1700
Zm00001d053378 CDS-3IBZ2    CCACCCTTTCTGAGGGAGGCGCCGAAACAAGCTTCTTCATGCACACGGTGGAGGATCCTCGCCACGTGGCGCCATCGGCCCGTGAAGGCCGGGTGCATGG   1700
Consensus                    ccaccctttctgagggaggcgccgaaacaagcttcttcatgcacacggtggaggatcctcgccacgtggcgccatcggcccgtgaaggccgggtgcatgg

Zm00001d053378 CDS-KW5G321   GGGCAACTTTATGCCTTTCATGCTGGATGGTGGTGTGGGACTCGGCAAGAAGCCGGCCAACAACGCTGACGCTAGTACCTCTGCCCCTCGCAGCTGGAAA   1800
Zm00001d053378 CDS-3IBZ2    GGGCAACTTTATGCCTTTCATGCTGGATGGTGGTGTGGGACTCGGCAAGAAGCCGGCCAACAACGCTGACGCTAGTACCTCTGCCCCTCGCAGCTGGAAA   1800
Consensus                    gggcaactttatgcctttcatgctggatggtggtgtgggactcggcaagaagccggccaacaacgctgacgctagtacctctgcccctcgcagctggaaa

Zm00001d053378 CDS-KW5G321   AATATGGAGAAGCCTGGTGCCTCTGCGCAGCGCAGCTGGCTGTGA   1845
Zm00001d053378 CDS-3IBZ2    AATATGGAGAAGCCTGGTGCCTCTGCGCAGCGCAGCTGGCTGTGA   1845
Consensus                    aatatggagaagcctggtgcctctgcgcagcgcagctggctgtga
```

I

```
Zm00001d053378 protein-KW5G321   MDPRHGRSAFTISFSNPNGFAYQCTPIPAVSYPATGSSQLDVLAPRGCKRKWTELALGLGDSSSSDSSKRSMGTGCTVSSAKGSDDVSCMDYDIGFELSL   100
Zm00001d053378 protein-3IBZ2    MDPRHGRSAFTISFSNPNGFAYQCTPIPAVSYPATGSSQLDVLAPRGCKRKWTELALGLGDSSSSDSSKRSMGTGCTVSSAKGSDDVSCMDYDIGFELSL   100
Consensus                        mdprhgrsaftisfsnpngfayqctpipavsypatgssqldvlaprgckrkwtelalglgdssssdsskrsmgtgctvssakgsddvscmdydigfelsl

Zm00001d053378 protein-KW5G321   GNEGTSRLCKQACDSTRTMEKPGLDLKLSLAPSQSDVTDADVIRSSAPQDMFVHHQYLMSSAPTVDEGSTSARRLSGGMVGSFLNQDGISLDQALPVNAN   200
Zm00001d053378 protein-3IBZ2    GNEGTSRLCKQACDSTRTMEKPGLDLKLSLAPSQSDVTDADVIRSSAPQDMFVHHQYLMSSAPTVDEGSTSARRLSGGMVGSFLNQDGISLDQALPVNAN   200
Consensus                        gnegtsrlckqacdstrtmekpgldlklslapsqsdvtdadvirssapqdmfvhhqylmssaptvdegstsarrlsggmvgsflnqdgisldqalpvnan

Zm00001d053378 protein-KW5G321   QVQGPAPSAPTVLQLPKSSAASSSGFVHPQQRSSITKICSHPGCAKGARGSSGRCIAHGGGRRCQKGGCSKGAEGKTIFCKAHGGGKRCEHLGCTKSAEG   300
Zm00001d053378 protein-3IBZ2    QVQGPAPSAPTVLQLPKSSAASSSGFVHPQQRSSITKICSHPGCAKGARGSSGRCIAHGGGRRCQKGGCSKGAEGKTIFCKAHGGGKRCEHLGCTKSAEG   300
Consensus                        qvqgpapsaptvlqlpkssaasssgfvhpqqrssitkicshpgcakgargssgrciahgggrrcqkggcskgaegktifckahgggkrcehlgctksaeg

Zm00001d053378 protein-KW5G321   RTDFCIAHGGGRRCSHEGCKRAARGKSGLCIKHGGGKRCEKPNCTKSAEGRSGMCIAHGGGRRCQYAGCGKGAQGSTSFCKAHGGGKRCTRPDCSKGAEG   400
Zm00001d053378 protein-3IBZ2    RTDFCIAHGGGRRCSHEGCKRAARGKSGLCIKHGGGKRCEKPNCTKSAEGRSGMCIAHGGGRRCQYAGCGKGAQGSTSFCKAHGGGKRCTRPDCSKGAEG   400
Consensus                        rtdfciahgggrrcshegckraargksglcikhgggkrcekpnctksaegrsgmciahgggrrcqyagcgkgaqgstsfckahgggkrctrpdcskgaeg

Zm00001d053378 protein-KW5G321   STAFCKAHGGGKRCSADGCTKSVHGGTQFCVAHGGGKRCVVEGCRKSARGRSDRCVGHGGGKRCHSAGCGKSAQGSTDFCKSHGGGRRCWWGHVGSGGAP   500
Zm00001d053378 protein-3IBZ2    STAFCKAHGGGKRCSADGCTKSVHGGTQFCVAHGGGKRCVVEGCRKSARGRSDRCVGHGGGKRCHSAGCGKSAQGSTDFCKSHGGGRRCWWGHVGSGGAP   500
Consensus                        stafckahgggkrcsadgctksvhggtqfcvahgggkrcvvegcrksargrsdrcvghgggkrchsagcgksaqgstdfckshgggrrcwwghvgsggap

Zm00001d053378 protein-KW5G321   CDRLARGRKGLCDRHNPLVDDNSVHGGVSFGGFSTLSEGGAETSFFMHTVEDPRHVAPSAREGRVHGGNFMPFMLDGGVGLGKKPANNADASTSAPRSWK   600
Zm00001d053378 protein-3IBZ2    CDRLARGRKGLCDRHNPLVDDNSVHGGVSFGGFSTLSEGGAETSFFMHTVEDPRHVAPSAREGRVHGGNFMPFMLDGGVGLGKKPANNADASTSAPRSWK   600
Consensus                        cdrlargrkglcdrhnplvddnsvhggvsfggfstlseggaetsffmhtvedprhvapsaregrvhggnfmpfmldggvglgkkpannadastsaprswk

Zm00001d053378 protein-KW5G321   NMEKPGASAQRSWL   614
Zm00001d053378 protein-3IBZ2    NMEKPGASAQRSWL   614
Consensus                        nmekpgasaqrswl
```

J

```
Zm00001d053393 Pro-KW5G321  CTAATTTAATGATACCTCCATCTAACTTTTTTCTCTGTAACTCATGAATGGATTCGTAGAGGATAATAACCCCTTCTAGGATATTAGGACCGGTCATAAA  100
Zm00001d053393 Pro-3IBZ2    CTAATTTAATGATACCTCCATCTAACTTTTTTCTCTGTAACTCATGAATGGATTCGTAGAGGATAATAACCCCTTCTAGGATATTAGGACCGGTCATAAA  100
Consensus                   ctaatttaatgatacctccatctaacttttttctctgtaactcatgaatggattcgtagaggataataaccccttctaggatattaggaccggtcataaa

Zm00001d053393 Pro-KW5G321  AGCTATCTACGAGGGACTGACAATTCTGTTTGCTACTAGGACTATTCTATTTGTGAGAACTTTAGTAATGATTTTGAACGACACATTAAGTAAGCAAATA  200
Zm00001d053393 Pro-3IBZ2    AGCTATCTACGAGGGACTGACAATTCTGTTTGCTACTAGGACTATTCTATTTGTGAGAACTTTAGTAATGATTTTGAACGACACATTAAGTAAGCAAATA  200
Consensus                   agctatctacgagggactgacaattctgtttgctactaggactattctatttgtgagaactttagtaatgattttgaacgacacattaagtaagcaaata

Zm00001d053393 Pro-KW5G321  GGTATGTAGTCATGGATTTTATTTGTGTTGTCTTTATGAACCGATAGGGAATTTTTATGAAAGTCCACAAACAACGCAAACAGATCAAACTTAATAATAT  300
Zm00001d053393 Pro-3IBZ2    GGTATGTAGTCATGGATTTTATTTGTGTTGTCTTTATGAACCGATAGGGAATTTTTATGAAAGTCCACAAACAACGCAAACAGATCAAACTTAATAATAT  300
Consensus                   ggtatgtagtcatggattttatttgtgttgtctttatgaaccgatagggaatttttatgaaagtccacaaacaacgcaaacagatcaaacttaataatat

Zm00001d053393 Pro-KW5G321  CCTAATAATTTCCCAAAACACTTGATAAAACGCAGCTGGAAACCATCTGGTCCAGGAGCTTCATTATGTTCCATATTAAATACTGCCTTTTTAACTTCTT  400
Zm00001d053393 Pro-3IBZ2    CCTAATAATTTCCCAAAACACTTGATAAAACGCAGCTGGAAACCATCTGGTCCAGGAGCTTCATTATGTTCCATATTAAATACTGCCTTTTTAACTTCTT  400
Consensus                   cctaataatttcccaaaacacttgataaaacgcagctggaaaccatctggtccaggagcttcattatgttccatattaaatactgcctttttaacttctt

Zm00001d053393 Pro-KW5G321  CTATAGTAAAATTAGCAGTTAGTATTTCATTCTCAGAGTCCGAAACTTGGGGGGGATATCATGAGTATTAGATTCGTCAAGTTCGATCAATGTTGTGTCT  500
Zm00001d053393 Pro-3IBZ2    CTATAGTAAAATTAGCAGTTAGTATTTCATTCTCAGAGTCCGAAACTTGGGGGGGATATCATGAGTATTAGATTCGTCAAGTTCGATCAATGTTGTGTCT  500
Consensus                   ctatagtaaaattagcagttagtatttcattctcagagtccgaaacttgggggggatatcatgagtattagattcgtcaagttcgatcaatgttgtgtct

Zm00001d053393 Pro-KW5G321  GATTTACCAAAGAGGTTTTTGTAATAACTTGTAATATGCTGTTTAAGCTCTTCCTCGTCTGATATACAAGAACCGGCATCATCCTCTAATGAATAAATCC  600
Zm00001d053393 Pro-3IBZ2    GATTTACCAAAGAGGTTTTTGTAATAACTTGTAATATGCTGTTTAAGCTCTTCCTCGTCTGATATACAAGAACCGGCATCATCCTCTAATGAATAAATCC  600
Consensus                   gatttaccaaagaggtttttgtaataacttgtaatatgctgtttaagctcttcctcgtctgatatacaagaaccggcatcatcctctaatgaataaatcc

Zm00001d053393 Pro-KW5G321  GTTGTCTCCGGTGTTTGCCATTAGCCACTAGATCATGAAAATATTTTGTATTATCATCCCCT.CGAGGAGATGTTTAACCATAGCACTCTAGAAGAGTCG  699
Zm00001d053393 Pro-3IBZ2    GTTGTCTCCGGTGTTTGCCATTAGCCACTAGATCATGAAAATATTTTGTATTATCATCCCTTTCGAGGAGATGATTGACCTTAACCCTCTGAAAGAGTCG  700
Consensus                   gttgtctccggtgtttgccattagccactagatcatgaaaatattttgtattatcatccc t cgaggagatg tt acc ta c ctct  aagagtcg

Zm00001d053393 Pro-KW5G321  AACTTCTTCCTCTCTCATAATACGAGTTAGCCTTTCATTATTAGCAAAAGCTTTAGTGTTTAACTCGTTAGACGATAAG..........CCGCCTTTT..  787
Zm00001d053393 Pro-3IBZ2    AACATCTTCCTCTCTCGTAAGACTGGCTATAATTTCCTT...AAAAAAAACTTTATCGTTCTCTTCGTTAGACGATAAGAAAGTAACTTCCGCCTTTTTA  797
Consensus                   aac tcttcctctctc taa ac  g ta   tttc tt   a  aaaa cttta  gtt    tcgttagacgataag          ccgcctttt

Zm00001d053393 Pro-KW5G321  ATCGAGTTCATCAATGAACTCAGAAAGTTGCTTCTTCTCCCTTTTTATTAACCCTGCTGATTGGCATCTATTATTGGTTCTATTGTTTCTTAATCGGGCT  887
Zm00001d053393 Pro-3IBZ2    ATGTTATTCATCAAACAACTCAAAAAGATGCTTCTTCTCCCTTTTTATTAACCCTGCTGATTGAAATCTATTATTGTCCCTATTGATTCTTACTCGTGAT  897
Consensus                   at    ttcatcaa  aactca aaag tgcttcttctccctttttattaaccctgctgattg  atctattattg   ctattg ttctta tcg g t

Zm00001d053393 Pro-KW5G321  TTTGTTGTTGGTTGTGCCGCGGAATCGTTCGTTCCTGT..ACGAAGAACATGATTCTTCTGCCAGGGTACTAGCATATTATTAGTCAAAACTGGCTACAT  985
Zm00001d053393 Pro-3IBZ2    TTTGTTGTATTATGGGCCGCATTGTCCCTCGTTCCTGTGTATGAGGAACATGATTCTTCTGCCAGG.TACTACCATATCATTAAACAAAACTGTTTACAT  996
Consensus                   tttgttgt    tg gccgc    tc  tcgttcctgt  a ga gaacatgattcttctgccagg tacta catat atta  caaaactg  tacat

Zm00001d053393 Pro-KW5G321  ACTTGACTTTAGAAACTGTATTGTCTTTAGGATATGACTGAAGAATGACGCTAGATGCTGTGGCCGAAAGCTTAATAACAAAACCTTCCCCAGACGGCTA  1085
Zm00001d053393 Pro-3IBZ2    ACTTGACTTGAAAAATTGTATTGTCTTTAGGATATGACTGAGAAATGACACTAGGTGCTGTT.TCGAAAGCTTAATAACAAAACCTTCCCCAGACGGTTA  1095
Consensus                   acttgactt a aaa tgtattgtctttaggatatgactga  aatgac ctag tgctgt   cgaaagcttaataacaaaaccttccccagacgg ta

Zm00001d053393 Pro-KW5G321  GGCTCCCGCTCAAATCAAGAGGGCCGGGTGGCAGTGGCACGCGCGCCTGTCGTTTGCCTGGCTCATTAGTCACCGTCAACAATGAAGCAGATCGTTTCGT  1185
Zm00001d053393 Pro-3IBZ2    GGCTCCCGCTCACATCAAGAGCTTCGCCAGACAGCGACGCTCGCGCCTGTCGTTTGCCTGGCTCATTAGTCACCGTCAACAATGAAGCAGATCGTTTCGT  1195
Consensus                   ggctcccgctca atcaagag   cg   g cag g c c cgcgcctgtcgtttgcctggctcattagtcaccgtcaacaatgaagcagatcgtttcgt

Zm00001d053393 Pro-KW5G321  GTAGCGTGGTCGTGGAATATTACTTGCCGTTGCCCGGCGCCTCAGGTAGTTTCTCCATGGCTTTTCTCGCACGGGGACGGGGCCCCGCAAGATACTTGGA  1285
Zm00001d053393 Pro-3IBZ2    GTAGCGTGGTCGTGGAATATTACTTGCCGTTGCCCGGCGCCTCAGGTAGTTTCTCCCTGGCTTTTCTCGCACGGGGACGGGGCCCCGCAAGATACTTGGA  1295
Consensus                   gtagcgtggtcgtggaatattacttgccgttgcccggcgcctcaggtagtttctcc tggcttttctcgcacggggacggggccccgcaagatacttgga

Zm00001d053393 Pro-KW5G321  TTAGCACAAAGGCAAGCTTTGGTATCTATCCTCGCTCTGAATGTGTGTGTGACAGGAGCAGGAGGGAGGGAGGGCGCACGGATGTTTTGACCAAGAGCTG  1385
Zm00001d053393 Pro-3IBZ2    TTAGCACAAAGGCAAGCTTTGGTATCTATCCTCGCTCTGAATGTGTGTGTGACAGGAGCAGGAGGGAGGGAGGGCGCACGGATGTTTTGACCAAGAGCTG  1395
Consensus                   ttagcacaaaggcaagctttggtatctatcctcgctctgaatgtgtgtgtgacaggagcaggagggagggagggcgcacggatgttttgaccaagagctg

Zm00001d053393 Pro-KW5G321  CCGGCATGGAAAAACGCGACGTCGGGTGGCCAGTCCCCGTCCCTAGTAGTCCAGTCCTGGAAGAGCCGGCTTCGGCGACCGCGACCACCGGACGACGAGA  1485
Zm00001d053393 Pro-3IBZ2    CCGGCATGGAAAAACGCGACGTCGGGTGGCCAGTCCCCGTCCCTAGTAGTCCAGTCCTGGAAGAGCCGGCTTCGGCGACCGCGACCACCGGACGACGAGA  1495
Consensus                   ccggcatggaaaaacgcgacgtcgggtggccagtccccgtccctagtagtccagtcctggaagagccggcttcggcgaccgcgaccaccggacgacgaga

Zm00001d053393 Pro-KW5G321  CGAGTCGAGGAGCGATGAATCCAAACGGACTGCCGTGCCATTTGACTAAAT.....CGTCGCGGACCCCACCGGCCCCGGAAGCTTCTTTCACACATGAC  1580
Zm00001d053393 Pro-3IBZ2    CGAGTCGAGGAGCGATGAATCCAAACGGACTGCCGTGCCATTTGACTAAATTAAATCGTCGCGCACCGCACCGGCCCCGTAATCTTCTTTCACACATGAC  1595
Consensus                   cgagtcgaggagcgatgaatccaaacggactgccgtgccatttgactaaat     cgtcgcg acc caccggccccg aa cttctttcacacatgac

Zm00001d053393 Pro-KW5G321  GTCAGACGAGGCGAGGTGCCTCTCTGCCGCCCTGCCCCGGAAGATTCGATTGAAGTATACTGCGTTACGTGCGCGCGTACTGCTGGA.TCATCCTCCTGT  1679
Zm00001d053393 Pro-3IBZ2    ACCAGACGAGGCGACTCTCCTCCCTGCCGCCCTGCCCCATAAGATTCGATTGAACTGAATTGCGTTACGTGCGCCCGCAGTGCTAGCCTCCTCCTGCTCT  1695
Consensus                     cagacgaggcga    cctc ctgccgccctgcccc  aagattcgattgaa t  a tgcgttacgtgcgc cg a tgct g  tc tcct ct t

Zm00001d053393 Pro-KW5G321  GTGCTGTCTGCGCACTTGTCGTCCGT.CCATTGAGCTGAGCGAGCCATCGATCCCGTGATCGCCGAA.CCCCCAGTTG.CCACCCT.CACCGGCTG.CCT  1774
Zm00001d053393 Pro-3IBZ2    GTGCTGGCAGTG.ACTTGCCGTCCCTTCCATTGAGCTGAAGCGA..AGCAATCCCGGGATCGCCGGATCCCCCAGTTGGCCACCCTTCACCGGTGGGCCG  1792
Consensus                   gtgctg c g g acttg cgtcc t ccattgagctga       a c atcccg gatcgccg a cccccagttg ccaccct caccgg  g cc

Zm00001d053393 Pro-KW5G321  GCCGTTCCCTCCG.CCTCTCAAATCC.AGTGCCGTGCGGGCGCGCGGCTCCCCAG....TTGGCAGTTGCCCAGCCCCCAGCAGC  1853
Zm00001d053393 Pro-3IBZ2    GCCGTTCCCTTCGGCCTCTCAAATCCCAGGCCCGTGCGGGCGCGCGGCTCCCCCGAGTGTGGAGAGTTGCCCAACCCCCCGCAGC  1877
Consensus                   gccgttccct cg cctctcaaatcc ag  ccgtgcgggcgcgcggctcccc g    t g  agttgccca ccccc gcagc
```

K

```
Zm00001d053393 CDS-KW5G321   ATGACTACTACGACCAAGAATCATAAGCAGCAGCAGCTGCTGCCCACGGCGGCGGCGGCCCCGCCTTGGCCCGTAAGGACTAAGCTCCTGCTCTGCGCCG   100
Zm00001d053393 CDS-3IBZ2     ATGACTACTACGACCAAGAATCATAAGCAGCAGCAGCTGCTGCCCACGGCGGCGGCGGCCCCGCCTTGGCCCGTAATGACTAAGCTCCTGCTCTGCGCCG   100
Consensus                    atgactactacgaccaagaatcataagcagcagcagctgctgcccacggcggcggcggccccgccttggcccgtaa gactaagctcctgctctgcgccg

Zm00001d053393 CDS-KW5G321   CCCTCGGCTTCGCGCTCGGCGTCGTGGCCACCGCGTCCCTCGTGAACTCCCCCTCCTACGCGACGTCGTCCGCCGCCGCCGCCGCGCTACGGGGAGGAGG   200
Zm00001d053393 CDS-3IBZ2     CCCTCGGCTTCTCGCTCGGCGTCGTGGCCACCGCGTCCCTCGTGAACTCCCCCTCCTACGCGACGTCGTCCGCCGCCGCCGCCGCGCTACAGGGAGGAGG   200
Consensus                    ccctcggcttc cgctcggcgtcgtggccaccgcgtccctcgtgaactcccccctcctacgcgacgtcgtccgccgccgccgccgcgctac gggaggagg

Zm00001d053393 CDS-KW5G321   AGGAGCGCTGGGCCTCGTCTTACCGCCGTCCGCCACCAGGAACGCCGCTCAGGACCGCCGCCCGCCGCCCAAACAAGCAGGCGCGGCACGGGCAACGGTC   300
Zm00001d053393 CDS-3IBZ2     AGGAGCGCTGGGCCTCGTCTTACCGCCGTCCGCCACCAGGAACGCCGCTCAGGACCGCCGCCCGCCGCCCAAACAAGCACGCGCGGCACGGGCAACGGTC   300
Consensus                    aggagcgctgggcctcgtcttaccgccgtccgccaccaggaacgccgctcaggaccgccgcccgccgcccaaacaagca gcgcggcacgggcaacggtc

Zm00001d053393 CDS-KW5G321   CGCCGGTCCCGACCGGAGCCGCCGGCTGCCACGGGCATCACTAGCGCTGCAGCCCCGCCGCCGTATGTTATTGGCGCCCAGGGACCAGCAGCGCCTAGTG   400
Zm00001d053393 CDS-3IBZ2     CGCCGGTCCCGACCGGAGCCGCCGGCTGCCACGGGCATCACTATCGCTGCAGCCCCGCCGCCGTATGTTATTGGCGCCCACGGACCAGCAGCGCCTACTG   400
Consensus                    cgccggtcccgaccggagccgccggctgccacgggcatcacta cgctgcagccccgccgccgtatgttattggcgccca ggaccagcagcgccta tg

Zm00001d053393 CDS-KW5G321   CCGTTGCCGCCTCCCGTGTCAATGGTGGTAGCATCGTCAGCGACAGCGACGGCGACGTCGACGGCGACGACGAGGAGCTCATGGCCAGGGCGGCGGCGTC   500
Zm00001d053393 CDS-3IBZ2     CCGTTGCCGCCTCCCGTGTCAATGGTGGTAGCATCGTCAGCGACAGCGACAGCGACATCGACAGCGACAACAAGGAGCTCATGGCCAGGGCGGCGGCGTC   500
Consensus                    ccgttgccgcctcccgtgtcaatggtggtagcatcgtcagcgacagcgac gcgac tcgac gcgac ac aggagctcatggccagggcggcggcgtc

Zm00001d053393 CDS-KW5G321   CGCGCCGCGGGAGGTGCCTGCCGGCTGCATGGTCCCCAGGGTGGCGTTCCTGTTCCTCACGCGCTGGGACC.TGCCCATGGCGCCGCTGTGGGATGACTT   599
Zm00001d053393 CDS-3IBZ2     CGCGCCGAGGGAGGTGCCTGCCGGCTGCATGGTCCCCAGGGTGGCGTTCCTGTTCCTCACTCGCTGGGACCCTGCCCATGGCGCCGCTGTGGGATGACTT   600
Consensus                    cgcgccg gggaggtgcctgccggctgcatggtccccagggtggcgttcctgttcctcac cgctgggacc tgcccatggcgccgctgtgggatgactt

Zm00001d053393 CDS-KW5G321   CTTCCGCGGCCACCGCGGCCTCTACAACGTCTACGTGCACTCCGACCCGGCGTTCAACGGCTCCGACCCGCCCGAGACCTCCGCCTTCTACCGCCGGAGG   699
Zm00001d053393 CDS-3IBZ2     CTTCCGCGGCCACCGCGGCCTCTACAACGTCTACGTGCACTCCGACCCGGCGTTCAACGGCTCCGACCCGCCCGAGACCTCCGCCTTCTACCGCCGGAGG   700
Consensus                    cttccgcggccaccgcggcctctacaacgtctacgtgcactccgacccggcgttcaacggctccgacccgcccgagacctccgccttctaccgccggagg

Zm00001d053393 CDS-KW5G321   ATCCCCAGCAAGGAGGTGAAATGGGGCGAGATCAGCATGGTGGAGGCGGAGCGCCGGCTTCTGGCGCACGCGCTGCTGGACGACCACTGCAACGCGCGCT   799
Zm00001d053393 CDS-3IBZ2     ATCCCCAGCAAGGAGGTGAAATGGGGCGAGATCAGCATGGTGGAGGCGGAGCGCCGGCTTCTGGCGCACGCGCTGCTGGACGACCACTGCAACGCGCGCT   800
Consensus                    atccccagcaaggaggtgaaatggggcgagatcagcatggtggaggcggagcgccggcttctggcgcacgcgctgctggacgaccactgcaacgcgcgct

Zm00001d053393 CDS-KW5G321   TCGTCCTCCTGTCGGAGTCGCACGTCCCGCTGTTCGATCTCCCCACGGTGCACTCCTACCTCGTCAACTCCACCAGGCTGTACCTCGAGTCCTACGACCA   899
Zm00001d053393 CDS-3IBZ2     TCGTCCTCCTGTCGGAGTCGCACGTCCCGCTGTTCGATCTCCCCACGGTGCACTCCTACCTCGTCAACTCCACCAGGCTGTACCTCGAGTCCTACGACCA   900
Consensus                    tcgtcctcctgtcggagtcgcacgtcccgctgttcgatctccccacggtgcactcctacctcgtcaactccaccaggctgtacctcgagtcctacgacca

Zm00001d053393 CDS-KW5G321   GCCAGGCGCGACGGGCCGCGGCCGCTACAACCGCCGCATGAGCCCCGTCGTGGCCGCGGGGCAGTGGCGCAAGGGCTCCCAGTGGTTCGACCTGGACCGG   999
Zm00001d053393 CDS-3IBZ2     GCCAGGCGCGACGGGCCGCGGCCGCTACAACCGCCGCATGAGCCCCGTCGTGGCCGCGGGGCAGTGGCGCAAGGGCTCCCAGTGGTTCGACCTGGACCGG   1000
Consensus                    gccaggcgcgacgggccgcggccgctacaaccgccgcatgagccccgtcgtggccgcggggcagtggcgcaagggctcccagtggttcgacctggaccgg

Zm00001d053393 CDS-KW5G321   GCCCTGGCCACAGACGTGGTCGCCGACCGCGTCTACTTCCCGCTCTTCCGCCGCTTCTGCCGCCGTCGCCACTGCTACGCCGACGAGCACTACCTGCCCA   1099
Zm00001d053393 CDS-3IBZ2     GCCCTGGCCACAGACGTGGTCGCCGACCGCGTCTACTTCCCGCTCTTCCGCCGCTTCTGCCGCCGTCGCCACTGCTACGCCGACGAGCACTACCTGCCCA   1100
Consensus                    gccctggccacagacgtggtcgccgaccgcgtctacttcccgctcttccgccgcttctgccgccgtcgccactgctacgccgacgagcactacctgccca

Zm00001d053393 CDS-KW5G321   CGCTCCTCAACATCGTCCGGCGCCCGTCGGCGGGGGCCAACCGGAGCCTCACGTGGGTCGACTGGTCCCACGGCGGCTGTCACCCCGCGCGGTTCACGAG   1199
Zm00001d053393 CDS-3IBZ2     CGCTCCTCAACATCGTCCGGCGCCCGTCGGCGGGGGCCAACCGGAGCCTCACGTGGGTCGACTGGTCCCACGGCGGCTGTCACCCCGCGCGGTTCACGAG   1200
Consensus                    cgctcctcaacatcgtccggcgcccgtcggcgggggccaaccggagcctcacgtgggtcgactggtcccacggcggctgtcaccccgcgcggttcacgag

Zm00001d053393 CDS-KW5G321   GATGGAGGTCACCGTCGACTTCCTCCGGTGGCTCAGGGAAGGCAGCACGTGCACGTATAACGGCAGGACCACCACAGTCTGCTTCCTTTTCGCCAGGAAG   1299
Zm00001d053393 CDS-3IBZ2     GATGGAGGTCACCGTCGACTTCCTCCGGTGGCTCAGGGAAGGCAGCACGTGCACGTATAACGGCAGGACCACCACAGTCTGCTTCCTGTTCGCCAGGAAG   1300
Consensus                    gatggaggtcaccgtcgacttcctccggtggctcagggaaggcagcacgtgcacgtataacggcaggaccaccacagtctgcttcct ttcgccaggaag

Zm00001d053393 CDS-KW5G321   TTCCTGCCGAACTCGCTCAC.CAGGTTCTTGAGGTTCGCGCCCAAGGTGATGGGGTTTGGTTAA   1362
Zm00001d053393 CDS-3IBZ2     TTCCTGCCGAACTCGCTCACACAGGTTCTTGAGGTTCGCGCCCAAGGTGATGGGGTTTGGTTAA   1364
Consensus                    ttcctgccgaactcgctcac caggttcttgaggttcgcgcccaaggtgatggggtttggttaa
```

L

```
Zm00001d053393 protein-KW5G321   MTTTTKNHKQQQLLPTAAAAPPWPVRTKLLLCAALGFALGVVATASLVNSPSYATSSAAAAALRGGGGALGLVLPPSATRNAAQDRRPPPKQAGAARATV   100
Zm00001d053393 protein-3IBZ2     MTTTTKNHKQQQLLPTAAAAPPWPVMTKLLLCAALGFSLGVVATASLVNSPSYATSSAAAAALQGGGGALGLVLPPSATRNAAQDRRPPPKQARAARATV   100
Consensus                        mttttknhkqqqllptaaaappwpv tklllcaalgf lgvvataslvnspsyatssaaaaal ggggalglvlppsatrnaaqdrrpppkqa aaratv

Zm00001d053393 protein-KW5G321   RRSRPEPPAATGITSAAAPPPYVIGAQGPAAPSAVAASRVNGGSIVSDSDGDVDGDDEELMARAAASAPREVPAGCMVPRVAFLFLTRWDLPMAPLWDDF   200
Zm00001d053393 protein-3IBZ2     RRSRPEPPAATGITIAAAPPPYVIGAHGPAAPTAVAASRVNGGSIVSDSDSDIDSDNKELMARAAASAPREVPAGCMVPRVAFLFLTRWDPAHGAAVG..   198
Consensus                        rrsrpeppaatgit aaapppyviga gpaap avaasrvnggsivsdsd d d d  elmaraaasaprevpagcmvprvaflfltrwd

Zm00001d053393 protein-KW5G321   FRGHRGLYNVYVHSDPAFNGSDPPETSAFYRRRIPSKEVKWGEISMVEAERRLLAHALLDDHCNARFVLLSESHVPLFDLPTVHSYLVNSTRLYLESYDQ   300
Zm00001d053393 protein-3IBZ2     ....................................................................................................   198
Consensus

Zm00001d053393 protein-KW5G321   PGATGRGRYNRRMSPVVAAGQWRKGSQWFDLDRALATDVVADRVYFPLFRRFCRRRHCYADEHYLPTLLNIVRRPSAGANRSLTWVDWSHGGCHPARFTR   400
Zm00001d053393 protein-3IBZ2     ....................................................................................................   198
Consensus

Zm00001d053393 protein-KW5G321   MEVTVDFLRWLREGSTCTYNGRTTTVCFLFARKFLPNSLTRFLRFAPKVMGFG   453
Zm00001d053393 protein-3IBZ2     .....................................................   198
Consensus
```

Supplementary Figure S1. sequence analysis of promoter, CDS and protein in KW5G321 and 3IBZ2. (A-C) Zm00001d053365 sequence analysis of promoter, CDS and protein in KW5G321 and 3IBZ2. (D-F) Zm00001d053366 sequence analysis of promoter, CDS and protein in KW5G321 and 3IBZ2. (G-I) Zm00001d053378 sequence analysis of promoter, CDS and protein in KW5G321 and 3IBZ2. (J-L) Zm00001d053393 sequence analysis of promoter, CDS and protein in KW5G321 and 3IBZ2.
